# Application of the STAT model for demand management to reduce specialist clinic waiting times: protocol for the WaitLESS stepped wedge cluster randomised controlled trial

**DOI:** 10.1136/bmjopen-2025-115253

**Published:** 2026-07-23

**Authors:** Katherine E Harding, Patrick Carney, Annie K Lewis, Julie Considine, Natasha Brusco, Luke Prendergast, David Snowdon, Mitchell N Sarkies, Philip Choi, Em Bould, Nicholas F Taylor

**Affiliations:** 1 Allied Health Clinical Research Office, Eastern Health, Box Hill, Victoria, Australia; 2 College of Science, Health and Engineering, La Trobe University, Melbourne, Victoria, Australia; 3 Department of Neurology, Eastern Health, Box Hill, Victoria, Australia; 4 Eastern Health Clinical School, Monash University, Melbourne, Victoria, Australia; 5 Florey Neuroscience Institutes, Parkville, Victoria, Australia; 6 School of Nursing and Midwifery; Centre for Quality and Patient Safety Research & Institute for Health Transformation, Deakin University, Geelong, Victoria, Australia; 7 Monash University, Rehabilitation, Ageing and Independent Living (RAIL) Research Centre, Victoria, Australia, Monash University, Melbourne, Victoria, Australia; 8 School of Health Sciences, The University of Sydney, Sydney, New South Wales, Australia

**Keywords:** Waiting lists, Health Services Accessibility, Organisation of health services, Delivery of Health Care, Integrated

## Abstract

**Introduction:**

Patients frequently face excessive waiting times for specialist medical outpatient clinics, negatively impacting health outcomes. Previous research has demonstrated that translation of demand-driven strategies incorporated within the evidence-based Specific Timely Assessment and Triage (STAT) model effectively reduces wait times in a range of healthcare settings. We outline the study protocol for a hybrid type II implementation effectiveness study, using a stepped wedge cluster randomised controlled trial design, to evaluate whether the STAT model can be implemented in specialist medical clinics and reduce waiting times.

**Methods:**

The Waiting List Evidence to Support Specialist clinics (WaitLESS) trial will implement STAT in eight clinical specialties (clusters) offering outpatient medical care at a metropolitan health network in Melbourne, Australia. After a 6 month pre-implementation period, clusters will be randomised to implement the STAT model, two clusters at a time at 3-month intervals, with a minimum 6-month follow-up period, following the STAT model’s five-step implementation process: (i) analyse historical clinic demand data, (ii) model supply/demand, (iii) implement target interventions to address existing backlogs, (iv) protect capacity for new patients to align with demand and (v) implement tailored strategies to enhance patient flow. Strategies will be implemented collaboratively by researchers and clinical teams. The trial will measure both effectiveness (reductions in waiting time and patients on the waiting list) and implementation (fidelity, adoption, acceptability, feasibility, appropriateness and cost) outcomes, using quantitative healthcare data and qualitative data from staff and patients.

**Analysis:**

Linear mixed-effects models will be used for analysis of the primary effectiveness outcome of waiting time from referral to first appointment, with time point and time since intervention as factor variables. Implementation outcomes will be analysed descriptively, with qualitative data mapped deductively to the Consolidated Framework for Implementation Research. A cost of implementation and cost-effectiveness analysis will be completed from the health service perspective.

**Trial registration number:**

ISRCTN15820171.

**STRENGTHS AND LIMITATIONS OF THIS STUDY**

- Robust design, using a stepped wedge cluster randomised controlled trial to test a service-level intervention to reduce waiting times in specialist medical outpatient services.
- Inclusion of implementation outcomes and a health economics analysis will provide valuable insights into how the model is implemented, reasons for success or failure, costs of implementation and cost-effectiveness.
- The intervention (the Specific Timely Assessment and Triage (STAT) model) brings together evidence-based principles into one structured, step-by-step package that is described using the Template for Intervention Description and Replication checklist for future replication.
- Lack of direct measurement of individual patient health outcomes could be considered a limitation. However, we will capture qualitative data on patient experience as well as health service use during the periods before and after the index outpatient appointment as a proxy indicator of changes in health status.

## Introduction

 Outpatient clinics providing access to medical specialists are a pivotal part of health systems around the world. These services enable diagnosis and specialist care, reduce reliance on inpatient services and support general practitioners to manage complex health conditions in the community.^1^ However, growing demand for specialist services, driven by an ageing population, increasing prevalence of chronic disease and consumer expectations, is driving long waiting lists for specialist clinics.^2–4^ While these delays are often viewed as an inevitable and intractable problem in the delivery of healthcare, they contribute to increased healthcare costs, psychological distress and poorer health outcomes.^5–7^

The mechanism of delivery of these services (described in this paper as ‘specialist clinics’) differs across countries and healthcare systems, spanning public and private funding systems. Publicly funded specialist clinics are often provided free or at low cost within universal public healthcare systems, such as those seen in Australia, the UK, New Zealand and Canada, but long wait times are common.^3 8^ However, access challenges for medical specialists have also been reported to be prevalent in systems with predominantly private funding, particularly for people experiencing social disadvantage.^9^

Despite evidence demonstrating that health service waiting lists can be reduced through service-level reform^10–14^ or policy interventions,^15^ long waiting times for specialist clinics remain a public concern.^16^ Data from Australia show half of patients wait more than 50 days across all specialties for a first appointment, and 10% of patients wait more than a year.^3 17^ The link between delays in access to care and poorer health outcomes has been well-established for a wide range of conditions.^7 18–21^ Furthermore, patients on outpatient waiting lists feel ‘forgotten’, are unable to plan for the future, experience deterioration in their health, are angry with the system and express a fear of ‘dying on the waiting list’.^5^

Strategies commonly used for managing waitlists and reducing waiting time in health services are often ineffective. Injections of resources to temporarily boost the supply of health services without a subsequent change in service delivery have only short-term effects, with waiting lists inevitably growing back over time.^4 14 22 23^ Long-term supply-side solutions are often hampered by workforce shortages and lack of funding. Triage systems are often used as a demand management tool but frequently lack reliability and contribute to inefficiencies by directing resources away from frontline care while doing little to solve the underlying problems contributing to the existence of the waiting list in the first place.^24^ Service providers are left with an impression that waiting lists are inevitable and give up on seeking solutions.^25^

However, there are a range of strategies that can be introduced by health services to reduce outpatient waiting times. Examples of principles of successful strategies include balancing supply and demand, improving efficiency of processes and protecting capacity for new patients and role substitution, in which alternative health providers undertake roles otherwise conducted by more specialised professionals.^10 11 13^ These strategies have been used in various combinations, often tailored to local context.^13^ This evidence is supported by consistent findings from a large number of studies, although limited by a predominance of single-group, observational study designs.^13^

The Specific Timely Assessment and Triage (STAT) model brings together several of these evidence-based principles into a step-by-step intervention and has led to sustainable reductions in wait times across a broad range of settings.^26–30^ STAT begins with analysis of supply and demand, followed by short-term, targeted interventions to reduce the existing waitlist and then protecting capacity specifically for new patients to enable access at the rate of arrival. New patients are booked in immediately for a first appointment without using a waiting list, and decisions about subsequent care are made by the treating clinician with consideration of patient need and service capacity. To date, the STAT model has been trialled in a range of allied health, community health and multi-disciplinary clinic settings, including a multi-site stepped wedge cluster randomised controlled trial resulting in a 34% reduction in waiting times (IRR 0.66, 95% CI 0.51 to 0.85).^27^ Specialist clinics share common features with other types of ambulatory services but also have unique challenges; they tend to see very large volumes of patients with mixed acuity, cross multiple funding systems and many face much larger backlogs of waiting patients than have been addressed in previous trials. Early success in a single pilot study in a single specialist outpatient clinic that almost eliminated a waiting list of 600 patients and sustained the service with minimal waiting lists over 4 years suggests it is feasible to apply the STAT model to specialist clinic settings, but further research is required to see whether it can be implemented at scale.^31 32^

This hybrid type II study using a stepped wedge cluster randomised controlled trial design aims to determine (i) whether the STAT model is effective in reducing waiting lists and wait times for specialist medical clinics and (ii) whether it can be successfully implemented across multiple services, including an embedded health economic analysis to determine the cost of implementation and cost-effectiveness of the model.

## Method

### Setting

The trial will take place at Eastern Health, a large network of metropolitan services in the eastern suburbs of Melbourne, Australia, providing publicly funded health services to a diverse community of approximately 892 000 people, including a high proportion (32%) of residents born overseas. Eastern Health offers a broad range of services, including emergency care, surgery, maternity, palliative care, mental health, drug and alcohol services, residential care and community health, delivered across three tertiary hospitals, two sub-acute inpatient services, a small community hospital, a day surgery centre and more than 30 community sites. The inclusion of a single health service could be seen as a limitation, but the size of the health network and its operation across multiple sites spanning a diverse geographical catchment provide confidence in the applicability of the findings to a broad range of health settings.

Eastern Health operates a comprehensive network of specialist medical clinics, providing expert care across a broad range of medical disciplines. Patients are typically referred to these clinics by general practitioners, specialists or hospital-based clinicians. Referrals are made electronically, then triaged by clinicians and assigned a priority category. Urgent referrals may be booked directly into appointments, while routine referrals are waitlisted until an appointment becomes available. Waiting times vary from weeks or months to several years.

### Design

We will conduct a hybrid type II effectiveness-implementation trial. Hybrid type II trials are designed to test both the clinical effectiveness of an intervention and the implementation strategy and can be effective in supporting more rapid research translation.^33^ This approach is well suited to this study, given that there is strong face validity for the intervention, strong indirect evidence and preliminary direct evidence of effectiveness, existing ‘implementation momentum’ (eg, stakeholder support), low risk and reasonable expectation that the implementation strategy is appropriate for this setting.^34^ The trial has been prospectively registered with the ISRCTN (UK National Clinical Study Register), registration number ISRCTN15820171 and reported in accordance with the STaRI checklist.

The design incorporates a stepped wedge cluster randomised controlled trial measuring both effectiveness and implementation outcomes with an embedded health economics evaluation to determine the cost of implementation and cost-effectiveness from the health service perspective. The health service perspective was selected as study findings will be primarily of interest to, and used by, providers of specialist clinics to support decision-making and appropriate allocation of funding.

**Figure 1:**
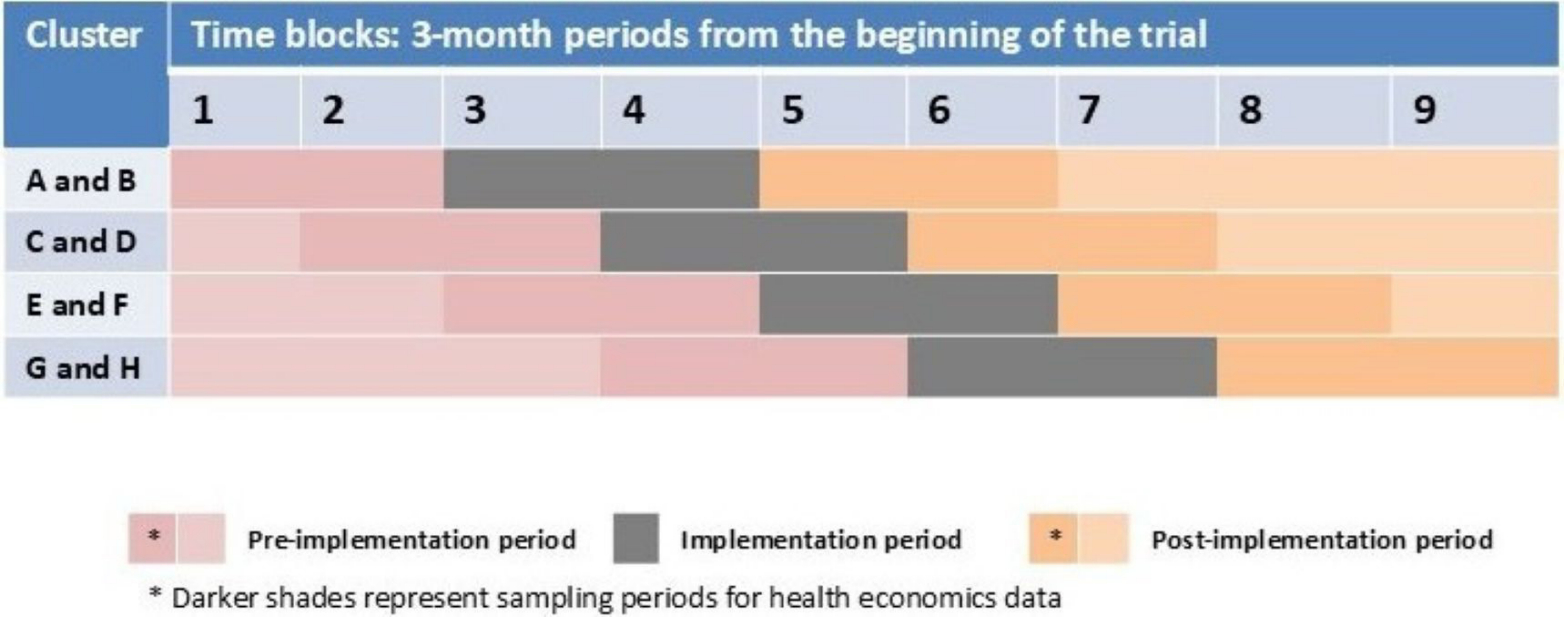
Implementation plan for a stepped wedge cluster randomised controlled trial.

The stepped wedge cluster randomised controlled trial (figure 1) will sequentially introduce the STAT intervention to clinics provided within eight speciality areas (clusters) in random order. Control data will be collected for 6 months (commencing 31 March 2025), after which the STAT model strategies will be introduced sequentially to two clusters within each step, with new steps commencing at 3-month intervals. There will be a 6-month period for implementation at each cluster, followed by commencement of collection of post-implementation data. The post-intervention data collection period for all clusters will conclude on 27 June 2027. While the design carries some risk of contamination and implementation fatigue due to the possibility of some of the same staff working across different clusters, this will be minimised through the inclusion of a diverse range of clinical specialties and a structured implementation process supported by project staff. Data will be collected for at least 6-month post-implementation at all clusters (figure 1). The primary effectiveness outcome will be a comparison of waiting (measured as time to first appointment and number of people on the waiting list) across the pre- and post-implementation periods. Implementation outcomes will be organised under six domains of acceptability, adoption, appropriateness, feasibility, fidelity and costs using a mixed-methods approach incorporating qualitative data and routinely collected healthcare data.

### Inclusion criteria

Included services (clusters) will be medical specialties (n=8) offering outpatient clinics at Eastern Health that have waiting lists with a minimum average of 12 weeks’ waiting time to first appointment and/or more than 200 people without appointments allocated. Given that referrals to a given specialty enter through a single waiting list but can then be distributed across multiple clinics (eg, operating at different locations across the health service), the clinics operating under each specialty will collectively form one cluster in the trial. Cluster selection will be decided in consultation with the medical directors of the clinics and senior leaders of the programme responsible for delivery of specialist medical clinics, based on (i) commitment from clinical leaders to initiate innovative changes to practice; (ii) potential for flexibility in service delivery (eg, variability in length or frequency of appointments, use of telehealth, involvement of nursing/allied health professionals in care) and (iii) the aim to include clusters across a spread of specialties.

Individual participants will include staff and patients who will contribute data to implementation outcomes. All staff working in the selected specialty areas during the study period (including medical staff, nursing and allied health staff, clerical staff and managers) will be invited to contribute to service reforms aligned with the intervention and be eligible to participate in focus groups. While some clinical specialties employ large numbers of sessional medical staff, we aim to include 4–8 participants per focus group for each clinic who are representative of key stakeholder groups and have detailed knowledge of clinic operations. For semi-structured interviews with patients, eligibility criteria will include the following: adult patients (or caregivers of children) who have been triaged as routine patients, recent attendance at a first appointment with a participating clinic; capacity to provide consent and sufficient oral communication capacity to participate in an interview (with an interpreter if required). Purposive sampling will be used with the aim of including a diverse range of participants.

### Randomisation and masking

The eight clusters will be randomly paired, and then the four pairs will be randomised for order of intervention. Randomisation will be undertaken using a random number sequence, computer-generated by an external clinical trial support service not involved in the study and blind to the identity of the participating medical specialties. Researchers and participants will not be informed which clusters will form the next intervention pair until 3 months prior to commencement of their implementation period. Clinic patients will not be aware of the study, and routinely collected data contributing to the primary outcome measure (referral and appointment dates used to calculate waiting time, patients allocated to the waiting list) are collected by a centralised data management service within the health organisation independent from the research team. Patients attending the service and the health service staff collecting the primary outcome can therefore be considered blinded to the intervention.

### Intervention and implementation strategy

The intervention will consist of a suite of service redesign initiatives based on the STAT model. During the implementation period, staff within each cluster will be trained in the STAT model in workshops of 1–4-hour duration (tailored for teams and individuals according to prior knowledge of STAT and role within the service, with options for flexible delivery), led by investigators Harding and Lewis. This training was developed at Eastern Health/La Trobe University and has been delivered to more than 1000 clinicians across Australia from more than 100 services over the past 4 years.^35^

Following training, the clinic staff will be guided through each of five steps of the STAT model: (i) analyse clinic supply and demand data, (ii) model supply and demand at the clinic, (iii) targeted interventions to address the existing backlog (using a comprehensive waiting list audit supplemented by targeted, short-term supply-side interventions), (iv) protect capacity for new patients to align with demand and (v) implement tailored strategies to enhance patient flow. Step 5 will incorporate enhanced clinic coordination, alongside other strategies that could include (but are not limited to) productivity improvements (such as reducing failure-to-attend rates), multi-disciplinary approaches to service delivery and partnerships with patients and primary care (table 1).

**Table 1:** Key WaitLESS intervention activities and corresponding resources

Key activities	Resources provided
**Staff training** in the principles of the STAT model	Four-hour workshop training provided by members of the research team
**STAT Step 1:** Retrospective collection and analysis of clinic data—typical supply and demand over past 2 years	Support from research team/project manager
**STAT Step 2:** Model supply and demand; determine how much capacity needs to be protected for new patients to maintain flow at the rate of demand	Support from research team/project manager
**STAT Step 3**: Address existing backlog. Example activities: Comprehensive waitlist audit: cleaning the list, including checking for errors and calling/messaging patients to determine need Short-term increase in supply Grouping similar patients for targeted interventions	Clerical assistance for auditing Some funding for additional short-term clinic supply
**STAT Step 4:** Redesign clinic templates to align with demand	Support from research team/project manager
**STAT Step 5:** Tailored interventions to enhance patient flow: Enhanced clinic coordination Other tailored strategies, such as multi-disciplinary approaches, partnerships with consumers and primary care	Part-time clinic coordinator during implementation and post-implementation periods Support from research team/project manager

STAT, Specific Timely Assessment and Triage; WaitLESS, Waiting List Evidence to Support Specialist.

Implementation of the intervention will be supported by the project team (including chief investigators based at the health network, as well as a project manager and research assistant), who will meet regularly with key stakeholders during the implementation period to facilitate the changes. Project funding will be used to employ a part-time clinic coordinator (12.5 hours per week) during the pre- and post-implementation period at each participating clinic (nursing or allied health background, as appropriate to the health conditions addressed by each clinic). Intervention elements are further described in table 2, using the Template for Intervention Description and Replication checklist.^36^

**Table 2:** Intervention described using the TIDieR checklist

Name	STAT model for reducing healthcare waiting times
Why/background	To address long waiting lists/waiting times for specialist clinic appointments.
What	The STAT model for managing demand for health services Five-step implementation process: Collect historical data on supply and demand for the services Analyse the supply/demand relationship—determine the throughput rate required to operate at the pace of demand Address the existing backlog using targeted strategies Comprehensive waiting list audits Short-term supply-side interventions Align clinic templates with demand and protect sufficient capacity for new patients Tailored strategies to enhance patient flow, including improved clinic coordination, efficiency measures, innovations to models of care and improved partnerships.
Who	Research team working in collaboration with clinical leaders, specialist medical staff, nursing/allied health staff and clerical support teams. Research team includes a project manager (0.6 EFT), research assistant/data manager (0.8 EFT) and two senior researchers (0.2 EFT each) actively contributing to the implementation process. A clinical coordinator employed (0.25 EFT) also employed at each clinic during the implementation/post-implementation periods to support implementation and embed changes
How	Research team analyses supply/demand data and provides training in the STAT model. Teams and researchers work collaboratively to work through steps 3–5 of the model and implement changes Regular meetings between researchers and clinic teams with support from clinic coordinator to implement process changes
Where	Large metropolitan health network in Melbourne Eight medical clinical specialty areas operating specialist medical clinics with waiting times of >3 months or with >200 patients waiting
When and how much	Six-month implementation period for each specialty Two new specialties commence at each step (every 3 months), four steps in total
Tailoring and modifications	Interventions tailored to each clinical specialty within the overall structure and principles of the STAT model
How well (fidelity)	Fidelity will be judged by the extent to which each of the STAT steps are completed Analysis of supply and demand completed, and the number of new appointments required to keep pace with demand calculated (STAT steps 1 and 2) Active measures taken to reduce the size of the existing waiting list Appointments for new patients protected in clinic schedules, based on demand Strategies implemented to enhance patient flow

EFT, equivalent full-time; STAT, Specific Timely Appointments for Triage; TIDieR, Template for Intervention Description and Replication.

### Data sources

Data will include routinely collected health data, qualitative data from staff and patients and cost and revenue data for the participating clinics.

#### Routinely collected healthcare data

Effectiveness outcomes will be drawn from routinely collected healthcare data, collected from the records of patients seen in the clinic during the pre-implementation and post-implementation periods (n>5600 patients) and de-identified for analysis. Clinic data will also be used for implementation outcomes, such as the number of patients scheduled and seen and failure to attend rates. Fields for datasets are included in online supplemental file A.

#### Qualitative data

Staff (estimate n=50–70) involved in the delivery of services at the participating clinics will be invited to participate in focus groups three times during the trial, aligned with the stages of the trial (pre-implementation, implementation and post-implementation). Patients who attend one of the eight participating clinics as a new patient during the pre-implementation or post-implementation periods (target n=48, three patients per cluster per time period) will be invited to participate in semi-structured interviews to explore changing perceptions of clinic experiences over time. Focus groups and interviews will be facilitated by members of the research team with experience in qualitative research methodology and will be recorded and transcribed. Participants will be provided with a copy of transcripts from interviews/focus groups and invited to provide comments or additional contributions (member checking). Written informed consent will be obtained from all focus group and interview participants (online supplemental file B).

#### Health economics data

During the 27-month study period, data for the cost of implementation analysis will be obtained from the following sources: (i) data extracts from clinic administrative and waiting list databases, (ii) audit of patient medical records and (iii) verbal reports obtained from clinic leaders through direct approach and semi-structured interviews. This costing data will be prospectively collected using a customised Excel spreadsheet at the end of each 3-month period (online supplemental file A details the data fields). The spreadsheet will capture 6-month pre-implementation, 6-month implementation and 6-month post-implementation of the STAT model (see figure 1). Cost-effectiveness analysis will include the cost of implementation data, as well as individual participant health service utilisation data, to determine the cost and tracking of patient waiting times to determine the effect.

### Outcome measures

#### Effectiveness outcomes

Quantitative outcomes will be obtained from routinely collected healthcare data and auditing of health service processes. The primary outcome measure for effectiveness will be waiting, measured as time from initial referral to first appointment and the number of people on the waiting list (measured weekly). Secondary outcomes include the number of emergency department presentations and days admitted to the health service during the 6 months prior to and following the first clinic assessment to determine whether there is a relationship between improved specialist clinic access and other health service utilisation (outpatient services, emergency presentations and health service admissions). These data will be collected for a subgroup of patients attending the clinic for the first time within two of the pre- and post-implementation time blocks (darker shades in figure 1). Factors that may be covariates that impact on waiting times will also be recorded (demographic data, diagnosis and referral source).

#### Implementation outcomes

Implementation measures are organised using the taxonomy of Proctor and colleagues, who described eight outcome measures for implementation research: acceptability, adoption, appropriateness, costs, feasibility, fidelity, penetration and sustainability.^37^ The current study includes measures within the first six of these outcome domains, acknowledging that the timelines planned for the current trial are likely to be insufficient to obtain a robust assessment of penetration and sustainability. These outcomes will be addressed in future studies.

Adoption outcomes will evaluate whether ‘action steps’ of the model have been implemented: targeted strategies to reduce the backlog of waiting patients, protected capacity for new patients based on demand and changes to models of care to enhance flow. Descriptions of backlog strategies and model of care changes will be obtained through staff focus groups and interviews with patients. The ratio of the number of new patients in the template to referrals received in the pre- and post-implementation periods will be used to determine whether clinics protected capacity to align with predicted demand.

Fidelity of the STAT model will be evaluated by measuring how well these action steps were completed. The outcomes of the backlog reduction process will be described using audit data (including the number of patients removed from this list and reasons why and the number scheduled for care). The average number of new referrals accepted and booked into appointments each week will be used to determine how well the clinics are able to align supply and demand in the pre- and post-implementation periods. Outcomes indicative of changes to models of care delivery at each cluster will be compared pre- and post-intervention, including: mode of care delivery (telehealth/face to face, type of practitioner), appointment outcomes (proportion discharged or booked for review), failure to attend rates, clinical patient contacts outside clinic times (eg, telephone follow-up, calls seeking advice) and the ratio of new to review patients in clinic schedules.

Acceptability, feasibility and appropriateness will be evaluated using qualitative data from staff and patients. Staff perspectives will be sought during the pre-implementation and implementation periods (perceptions of current state, views about the intervention and challenges/enablers for implementation) as well as 6 months after the commencement of the post-implementation period (perceived success or failure of the intervention, impact on patient care, staff workloads and job satisfaction and reflections on feasibility and appropriateness of the STAT model for specialist clinics). Patient perspectives of the clinic service will be sought from samples of patients who attend during the pre- and post-implementation periods.

The total cost of implementation will be considered in two parts: (i) Cost of initial backlog reduction (auditing and short-term additional supply) per patient removed from the waiting list and (ii) total cost of clinic operations during the pre- and post-implementation periods. The cost-effectiveness of the STAT programme will determine the incremental cost per percentage of urgent referrals seen within 30 days and routine referrals seen within 90 days, comparing usual care conditions to intervention conditions. Individual patient health service utilisation cost (outpatient services, emergency presentations and health service admissions) will be collected for the 6 months prior to their first clinic appointment and the first 6 months following this appointment, for patients under usual care conditions and for patients under intervention conditions (estimated n=800).

Outcome measures across all aspects of the trial are summarised in table 3.

**Table 3:** Summary of outcome measures and data sources

	Routinely collected data	Consumer interviews	Staff focus groups	Prospective audits
**Effectiveness outcomes**				
Primary outcomes:				
Time from referral to first appointment	✓			
Proportion of patients seen within thresholds	✓			
Number of patients on the waiting list	✓			
Covariates:				
Demographic data	✓			
Referral data	✓			
Secondary outcomes:				
Health service use (outpatient services, emergency presentations and health service admissions)	✓			
**Implementation outcomes**				
**Adoption**				
Evidence of targeted backlog reduction activity				
Strategies implemented			✓	
Evidence of protected capacity for new patients				
Number of weekly appointments scheduled for new and review patients	✓			
Evidence of changes to model of care				
Staff perceptions of service changes			✓	
Patient experience of services		✓		
**Fidelity**				
Descriptive analysis of backlog reduction activity				
Number of patients removed from list				✓
Reasons for removal (appointment previously provided, service not needed, unable to contact)				✓
Number of patients scheduled for care				✓
Ratio of expected demand to new patient appointments booked	✓			
Indicators of change in care delivery				
Mode of delivery (face to face, telehealth)	✓			
Number of appointments scheduled by professional type (medical, nurse, allied health)				
Appointment outcomes (proportion rebooked/discharged)	✓			
Attendance (expressed as proportion failed to attend)	✓			
Ratio of new to review patients scheduled	✓			
Contacts with clinic staff outside of the allocated clinic appointments (eg, telephone advice and follow-up)				✓
**Acceptability, feasibility and appropriateness**				
Patient perceptions		✓		
Staff perceptions			✓	
**Cost**				
Costs to health service:				
Backlog reduction strategies				✓
Service delivery	✓			
Revenue	✓			

### Sample size

A minimum average of 400 referrals is expected to be received for each included specialty annually (100 per quarter), resulting in at least 700 patients across the pre- and post-implementation periods for each cluster (minimum total sample, n=5600). With four steps and eight clusters (two clusters per step), this stepped wedge design will be well powered to detect even small-to-moderate effect sizes. For a mean difference of 0.25 SD in waiting time and a conservative ICC of 0.1, with at least 60 referrals per cluster per 3-month time period (which is less than the pro-rata expected number annually), this design is powered at approximately 90% to sufficiently detect a difference at a 5% level of significance.^38 39^ Again, under conservative assumptions, power for detecting significant differences for our secondary outcomes (eg, an increase in the proportion of urgent patients seen within 30 days from 80 to 90%) exceeds 90%. Compared with our findings from STAT implementation in community outpatient services,^27^ our power and sample size assumptions are chosen conservatively to more than compensate for additional heterogeneity or other differences in the specialised clinic setting.

The sample size for quantitative implementation outcomes is based on the duration of the intervention and length of the waiting lists of the included services. The sample size for the qualitative analysis is considered feasible and expected to be sufficient to reach information power.

### Analysis

Separate analyses will be undertaken for each of the study objectives.

#### Effectiveness

Linear mixed effects models will be used to analyse data related to the primary outcome with time-point (time since trial start) and time since intervention as factor variables. This allows for the expected nonlinear change over time, as well as an analysis of persistence of intervention and time-averaged intervention effects following the service redesign, consistent with recommendations.^40^ Generalised linear mixed effects models will be used to analyse the secondary outcomes (binomial for proportions and negative binomial for counts to allow for over-dispersion). A random effect for the clinic will be used for all models to account for clustering. Visual diagnostics will be used to assess model assumption validity, and transformations of outcomes or robust estimators will be used as needed. Adjusting for and analysing the association between covariates (eg, referral source and demographic data, including potential indicators of disadvantage such as need for interpreters) and outcomes will also be completed by including these in the mixed-effects models as predictors. Given the small number of clusters, the Kenward-Roger small-sample correction will be used for the df for inference of the fixed effects.^41^

In the event of any system-wide (eg, pandemic) and within-cluster notable disruptions (eg, staffing issues) that are not adequately captured by the baseline random-effects structure and residual variation, a disruption indicator variable will be used for the disrupted time period. This model of adjustment to disruption can also be included as an indicator within affected clusters only. As an additional sensitivity analysis, within-cluster time-series analyses will be conducted to account for localised temporal disruptions while assessing the persistence of intervention effects. R V.4.4.1 (or a newer version if available) will be used for the analyses.

#### Implementation

Quantitative implementation outcomes, including the outcome of the waiting list audits, the ratio of demand to clinic capacity for new patients and indicators of change in patient flow, will be analysed descriptively. Qualitative data generated from staff will be mapped deductively to the domains of the Consolidated Framework for Implementation Research (CFIR). Rating rules for the CFIR and a cross-comparison analysis will be used to compare the experiences of different clinic teams.^42^ This method of analysis allows each comment made by participants to be coded in relation to its relevance to a subdomain of the CFIR and rated as ± according to whether the comment suggests a positive or negative influence of the factor on implementation.

Qualitative data generated from patients will be analysed using a thematic matrix analysis.^43^ Initially, the interview transcripts will be coded inductively line by line, then codes will be used to identify themes and subthemes. These themes will then be organised into a matrix to compare perceptions of patients who attended the clinic during the pre-implementation period and those who attended during the post-implementation period.

A cost of implementation and cost-effectiveness analysis will be completed from a health service perspective. A priori subgroup analysis of clinic patient types (urgent vs routine) will be completed as part of the economic evaluation. The cost of implementation will include two parts: (1) the cost of initial backlog reduction and (2) the cost of clinic operations. The cost of initial backlog reduction will be calculated by dividing the total cost by the incremental difference in the number of patients on the waitlist at the end of the pre-implementation period and the number of patients on the waitlist at 30-day post-implementation. The total cost of clinic operations will be calculated using quarterly audits throughout the trial to record all usual care costs (usual care staffing, capital and overhead costs) as well as any additional investment associated with the intervention, less the income derived from activity-based funding received by each cluster. A unit cost will be calculated by dividing the total cost for each cluster in each 3-month period by the number of new patients who receive a first appointment with a clinic within that cluster during that time. Each of these costs will be reported as a cost per cluster and as mean costs (with SD) across clinics. Costing at the service level will enable comparative efficiency measures such as the ratio of inputs to service output. The cost of implementation analysis will be presented as an overall cluster cost, as well as a cluster cost for the pre-implementation period and as a cluster cost for the post-implementation period.

To establish the incremental cost-effectiveness ratio (ICER), pre-implementation to post-implementation, cost differences will be divided by the percentage difference of urgent and routine referrals seen within 30 and 90 days, respectively, to determine the cost per 1% increase in the proportion of patients seen within these thresholds. ICERs will be determined by bootstrapping individual cost and effect data 5000 times, and this will be presented as an ICER value with 95% CIs and visually presented on a cost-effectiveness plane.

All cost data will be inflated to AUD$ 2026/2027 (final year of data collection) using the consumer price index and will take a health service perspective.^44^ Due to the nature of the economic evaluation planned, economic modelling will not be required. A discount rate will not be applied due to the limited time horizon and future economic impacts not being considered.

### Patient and public involvement

This proposal was developed through a partnership between Eastern Health, La Trobe University and the Victorian Department of Health. The research team includes ten chief investigators and four associate investigators, including two consumer representatives. Consumer researchers are active collaborators within the team and will be contributory authors to primary papers. In addition, consumer advisory panels will be convened during the implementation phase of the trial if there is a need to involve a wider spectrum of consumer voices in the development of ideas to enhance the flow of patients through clinic services.

### Trial governance

The research team oversees the implementation of the trial and manages the budget and analysis and reporting of results. The governance structure also includes a steering committee with representatives from the project team, partner organisations and consumer representatives that monitors implementation, provides strategic support and assists with the identification and management of risks. During time period five (see figure 1), a data safety and monitoring committee will review the implementation of the intervention at clusters A and B, including access to routine hospital reporting related to waitlists and waiting times and reports from key stakeholders (medical leads and service managers). Any indications of unintended adverse consequences arising from the intervention at these first two clusters will be reported to the steering committee and research team to consider implications for continuation of the trial.

## Ethics and dissemination

### Ethics

This project has been approved by the Eastern Health Human Research Ethics Committee (E24-020-111382, online supplemental file C), with acknowledgement/acceptance from affiliated universities involved in the trial in accordance with local policies.

### Dissemination

A key objective of the Waiting List Evidence to Support Specialist study is to successfully translate study findings into policy and practice. Specific strategies for translation will be influenced by the trial findings and developed in consultation with other consumer investigators and consumer advisory panels, the clinicians involved in delivery of the intervention and a broader consultative process inclusive of policy makers and service providers from specialist clinics. These strategies will be designed with reference to the Practical Implementation Sustainability Model (PRISM)^45^ and are planned to include wide dissemination strategies targeting policy makers and providers of specialist clinics. In addition, we will disseminate findings of the study through published manuscripts, conference presentations and the STAT website (www.thestatmodel.com). Trial data will be made available through La Trobe University's Institutional Repository (OPAL) in line with Australian National Health and Medical Research Council (NHMRC) policies.

## Supplementary material

10.1136/bmjopen-2025-115253 online supplemental file 1

10.1136/bmjopen-2025-115253 online supplemental file 2

10.1136/bmjopen-2025-115253 online supplemental file 3
