# Supplementary material for: Application of the STAT model for demand management to reduce specialist clinic waiting times: protocol for the WaitLESS stepped wedge cluster randomised controlled trial
Source: BMJ Open. 2026 Jul 23;16(7):e115253. doi: 10.1136/bmjopen-2025-115253 (PMC13404491; doi:10.1136/bmjopen-2025-115253)
Supplement: online supplemental file 1 [file bmjopen-16-7-s001.docx]

# Supplementary file A: Datasets

### Dataset 1: Waiting time data (effectiveness primary outcome and co-variates)

**Purpose:** To obtain the primary outcome for the trial (waiting time from referral to first appointment), as well as demographic, referral and appointment data to describe the sample and co-variates that may impact on waiting time for the clinic.

**Inclusion criteria:** All patients scheduled to attend an appointment for the first time at each participating clinic during the control and intervention periods at each clinic (refer to Fig. 1).

**Source:** Report drawing data from IPM and/or EMR generated by the Eastern Health Data Analytics team.

**Estimated number of records:** 5,600 (based on an average referral rate of 400 patients per clinic per year, or 100 per quarter).

Variables for inclusion:

- Clinic attended
  - Demographic data (age, gender, interpreter required, postcode)
  - Referral data (referral source, referral date, triage category, reason for referral, primary medical condition)
  - Appointment data (date of appointment, attended/not attended, rebooked/discharged, face to face/telehealth).

### Dataset 2: Health service use (effectiveness – secondary outcome)

**Purpose:** To obtain data for the secondary outcome of health service use to determine whether there is an association between improved specialist clinic access and use of other health services. This dataset will also be used to calculate costs associated with health service use for the health economics analysis.

**Source:** Report generated by the Eastern Health Data Analytics team.

**Inclusion Criteria:** All patients who attend their first appointment with an included clinic in either the last two quarters of the control period or first two quarters of the intervention period (subgroup of the patients included in dataset 1 represented by the striped blocks in Fig. 1).

Estimated number of records: 3,200

**Variables for inclusion:** Number of emergency department presentations, number of separate admissions, and total number of bed days admitted to Eastern Health during the six-months prior to the index clinic appointment and six-months following the appointment for each patient.

### Dataset 3: Service level outcomes: Number of patients on the waiting lists (effectiveness outcome) and implementation outcomes (adoption, fidelity)

**Purpose:** To measure clinic activity during the pre- and post-implementation periods at each clinic to evaluate implementation outcomes.

**Source:** Eastern Health Insight report (OPATOR13S) supplemented with manual prospective auditing of clinic schedules within the IPM database and direct observation of clinic activity.

**Inclusion criteria:** All clinic sessions conducted by each of the 8 participating clinics during the control and intervention periods (refer to Fig. 1).

**Estimated number of records:** For this dataset, each week of activity for each clinic will be considered as one record, making a total of 728 records (91 per clinic).

**Variables for inclusion:** For each week of activity, the following data will be prospectively collected:

- Number of appointments scheduled
- Number of appointments attended
- Proportion of new vs review appointments scheduled
- Proportion of appointments delivered by different professionals (doctor, nurse or allied health)
- Clinical patient contacts with the clinic coordinator outside clinic times (e.g. telephone advice and follow up, prospectively audited in the intervention period only)
- Number of patients on the waiting list

No direct patient data will be recorded in this dataset, only counts of appointments/interactions.

### Dataset 4: The outcomes of the waiting list audits conducted at each clinic (Implementation – fidelity)

**Purpose:** One aspect of the intervention involves a comprehensive audit of the waiting list at each site. Data will be prospectively collected during this audit to inform outcomes for the process evaluation.

**Source:** A list of patients on the waiting list will be initially generated from IPM by a clerical staff member associated with the project team, including the demographic variables outlined below. Other data will be collected and recorded directly by the clerical staff conducting the audit during the audit process.

**Inclusion criteria:** All patients on the waiting list (referred but do not yet have an appointment scheduled) at the beginning of the implementation period at each clinic.

**Estimated number of records:** 3,200 (based on an average waiting list of 400 patients at each clinic).

Variables for inclusion:

- - **Demographic variables** (UR, name, age, sex, source of referral, referral date, referral reason, primary medical condition)
  - **Audit outcome** (removed from list, requires appointment)
  - **If removed**: Reason for removal from the list (unable to contact, service no longer required, already seen, multiple failures to attend, died)
  - **If requiring appointment**: Audit outcome (return to waiting list, appointment scheduled)

The time taken for auditing will also be recorded by the auditors to contribute to the health economic analysis.

### Dataset 5: Clinic costs (Implementation outcome - cost)

We will calculate total costs of providing the clinic service using 3-monthly audits throughout the trial to record all usual care costs (usual care staffing, capital and overhead costs) as well as any additional investment associated with the intervention, less the income derived from activity-based funding received by the clinic. These data will be collected by the project manager in consultation with the specialist clinics management team with assistance from the Eastern Health data analytics and finance teams as required.

| **Cost category** | **Item** | **Unit** | **Costs allocated** |
| --- | --- | --- | --- |
| **Clinic Operations** | | | |
| Clinic rooms | Rooms used by the clinic | Number of rooms/hours used | Square metre infrastructure cost x rooms x hours |
|  | Hours per session |  |  |
| SESSIONAL HOURS: Number of clinic templates that ran in reporting period | CONSULTANT | Actual number of hours | Staff cost per hour x total hours |
|  | REGISTRAR |  |  |
|  | NURSE (sessional/templated) |  |  |
|  | ALLIED HEALTH (sessional/templated) |  |  |
| NON-SESSIONAL HOURS: Average per week this quarter | Clerical staff (bookings) | Estimated hours – average/week multiplied by number of weeks in reporting period | Staff cost per hour x estimated hours |
|  | Clerical staff (reception) |  |  |
|  | Nurse (non-sessional) |  |  |
|  | WaitLESS Clinic Coordinator |  |  |
|  | Consultant - average number of salaried hours |  |  |
|  | Registrar - average number of salaried hours |  |  |
|  | Others (specify) |  |  |

| STAT Implementation costs | | | |
| --- | --- | --- | --- |
| Analysis of clinic supply/demand data | WaitLESS Clinic Coordinator | Estimated hours | Staff cost per hour x estimated hours |
|  | WaitLESS Project officer |  |  |
| Other implementation activities  Training | All staff involved,  Itemised by professional classification/salary | Actual hours | Cost per hour x hours for each classification |
| Meetings |  |  |  |
| Waitlist auditing |  |  |  |
| Extra clinics for backlog reduction |  |  |  |
| Planning and implementing new strategies to improve patient flow |  |  |  |
| Consumer consultation | Itemised costs (eg catering, gift vouchers) | Per unit | Cost x unit |
| Consumables | Itemised cost | Per unit | Cost x unit |
