# Supplementary material for: Application of the STAT model for demand management to reduce specialist clinic waiting times: protocol for the WaitLESS stepped wedge cluster randomised controlled trial
Source: BMJ Open. 2026 Jul 23;16(7):e115253. doi: 10.1136/bmjopen-2025-115253 (PMC13404491; doi:10.1136/bmjopen-2025-115253)
Supplement: online supplemental file 2 [file bmjopen-16-7-s002.docx]

**Supplementary file B**

**Participant information and consent forms**

1. Staff participants in focus groups

2. Consumer participants in interviews

**Participant Information Sheet/Consent Form**

**Eastern Health staff participants – focus groups**

| **Title** | **WaitLESS: Applying data-driven, evidence-based**  **principles to reduce waiting lists in specialist clinics** |
| --- | --- |
| **Short Title** | **WaitLESS** |
| **Protocol Number** | 2 (04/12/2024) |
| **Project Sponsor** | La Trobe University |
| **Principal Investigator** | Professor Katherine Harding (Eastern Health, La Trobe University) |
| **Associate Investigators** | Associate Professor Patrick Carney (Eastern Health)  Professor Nicholas Taylor (Eastern Health, La Trobe University)  Dr Annie Lewis (Eastern Health, La Trobe University)  Professor Julie Considine (Deakin University)  Associate Professor Natasha Brusco (Monash University)  Professor Luke Prendergast (La Trobe University)  Dr David Snowdon (Peninsula Health, La Trobe University)  Dr Mitchell Sarkies (University of Sydney)  Dr Philip Choi (Eastern Health, Monash University) |
| **Location** | Eastern Health |

**Part 1**  **What does my participation involve?**

1. **Introduction**

You are invited to take part in this research project, which is called *“****WaitLESS: Applying data-driven, evidence-based*** ***principles to reduce waiting lists in specialist clinics.”*** You have been invited because you are an employee of Eastern Health, in a role that is relevant to delivery of services in one or more of the specialist medical clinics involved in the WaitLESS project.

This Participant Information Sheet/Consent Form tells you about the research project. It explains the processes involved with taking part. Knowing what is involved will help you decide if you want to take part in the research.

Please read this information carefully. Ask questions about anything that you don’t understand or want to know more about. Before deciding whether or not to take part, you might want to talk about it with colleagues or your manager.

Participation in this research is voluntary. If you don’t wish to take part, you don’t have to.

If you decide you want to take part in the research project, you will be asked to sign the consent section. By signing it you are telling us that you:

• Understand what you have read

• Consent to take part in the research project

• Consent to be involved in the research described

• Consent to the use of your personal and health information as described.

**2 What is the purpose of this research?**

Wait lists are a widespread problem in health services. The WaitLESS project aims to demonstrate how waiting lists for specialist clinics can be substantially reduced at scale, using practical, cost-effective strategies that are not reliant on additional medical specialists.

This qualitative study forms one aspect of the WaitLESS project, and is intended to formalise the collection, recording and reporting of qualitative data regarding staff experience. These data aim to answer questions about how and why the results of the project come about, and the process of implementing the changes.

**3** **What does participation in this research involve?**

The redesign of clinic processes and implementation of strategies to reduce waiting times will occur in consultation with staff and managers at the affected clinics. As a staff member at a participating clinic, you will have the opportunity to contribute to this process as part of your usual role and this will not be impacted by whether or not you agree to be a research participant in this qualitative arm of the WaitLESS project.

If you agree to participate, you will be invited to take part in three focus groups within the next 12 months. The groups will be made up of staff members involved with the delivery of medical specialist clinic services at Eastern Health involved in the WaitLESS project, including medical staff, other clinical staff involved in the clinic (such as nursing or allied health staff) and clerical support staff. The focus groups are different from your usual team meetings in that the content will be recorded and analysed using formal research methods.

The series of three focus groups will be conducted at different times in the WaitLESS project:

1. Before implementing strategies to reduce waitlists (to determine the perceived strengths, challenges, or barriers to implementing the strategies proposed, and to understand your perceptions regarding the consultation and development process to date);
2. During the implementation period (to understand your perceptions of the intervention during the initial stages of implementation, including challenges/enablers of implementation); and
3. Six months after commencing waitlist reduction strategies (to understand the perceived success or failure of the intervention, impact on patient care, staff workloads and job satisfaction).

The sessions will take between 30-60 minutes and be guided by a member of the research team.

Prior to commencing the first focus group we will ask you to complete a short questionnaire to collect some basic demographic information about you and your role at Eastern Health, for the purpose of describing our participants. This may include questions about your age and gender, your professional group (for example, administrative, medical, allied health, or nursing), the clinic you are currently employed at, and how long you have been employed at this clinic, or similar clinics. The questionnaire has been designed using categorical options for you to select from, such as age group and professional group, to minimise the possibility of you being personally identified from your demographic information. To further maintain your anonymity, all data provided will be de-identified and aggregated with the responses from other focus group participants prior to reporting this information.

This research project has been designed to make sure the researchers interpret the results in a fair and appropriate way and avoids researchers or participants jumping to conclusions. The focus groups will be audio recorded. After the focus groups, we will transcribe the audio recordings. The audio recordings will be deleted once transcription has been completed. You will receive a copy of the transcript from the focus groups that you attend. You will then have an opportunity to comment on the transcript, and to add additional thoughts on the topic that you would like to contribute.

There are no costs associated with participating in this research project. Focus groups will be scheduled within your usual work hours at Eastern Health, during which time you will be paid your usual salary.

**4** **Other relevant information about the research project**

We aim to determine whether the WaitLESS strategies are effective in improving timely access to outpatient appointments. To do this, data will be collected for six months prior to, and at least six months following, implementation of wait list reduction strategies. The main outcome of interest will be patient waiting time between referral to a specialist clinic and first appointment. Other service, patient, process, and cost outcomes will be collected. The focus groups will complement this data by assisting us to understand how implementation occurred from the perspective of the staff involved in the specialist clinics.

**5 Do I have to take part in this research project?**

Participation in any research project is voluntary. If you do not wish to take part in these focus groups, you do not have to. If you decide to take part and later change your mind, you are free to withdraw from the project at any stage.

If you do decide to take part, you will need to provide an electronic signature on this form to indicate your consent. You will then be given the option to print a copy of this Participant Information and Consent Form or have one sent to you by email for your records.

Your decision whether to take part or not to take part, or to take part and then withdraw, will not affect your employment at Eastern Health. Your decision on whether or not to take part in the focus groups will also not affect your participation in general staff consultations that will form part of the planning and implementation of WaitLESS strategies.

**6** **What are the possible benefits of taking part?**

There is no guarantee of a benefit to you from your participation in this research. However, these focus groups will provide an opportunity for you to tell us about what is working well, the barriers that you face and problems that need to be addressed in relation to implementation of these strategies to improve access. This information has the potential to directly lead to interventions that will improve your workflow and the care your patients receive.

We do expect this project to have significant benefits for the broader community. We hope to learn whether the WaitLESS strategies will reduce wait times in specialist clinics at Eastern Health and whether the findings could be further generalised. If successful, the project may lead to direct benefits for future patients at this and other health services.

**7** **What are the possible risks and disadvantages of taking part?**

There are no foreseeable risks to you in taking part in this project. In the unlikely event that you feel that any of the questions or discussions are stressful or upsetting, you may stop immediately. If you experience any distress associated with your participation in the research project, the research team will be able to arrange appropriate support.

While we will do all that we can to maintain confidentiality and anonymity of participants and will reinforce the importance of confidentiality during the session, we cannot guarantee that the content of the discussions will not be shared by other participants beyond the group. Given that each focus group may include some individuals with specific roles or skill sets, participants should be aware that it may be possible for those familiar with the service to guess the identity of a person based on the data reported.

**8 What if I withdraw from this research project?**

If you do consent to participate, you may withdraw from the focus group series at any time. If you decide to withdraw, please notify the project manager or a member of the research team. You will be provided with and asked to complete and sign a ‘Withdrawal of Consent’ form.

If you decide to leave the research project, the researchers will not collect additional personal information from you. You should be aware that data collected up to the time you withdraw will form part of the research project results, as data from a focus group transcript cannot be easily removed without affecting the continuity of the discussion. However, if this situation should arise, we will avoid reporting any direct quotes from you in published materials or project reports.

**9 Could this research project be stopped unexpectedly?**

It is possible, but unlikely, that this research project may be stopped unexpectedly. For example, the project may be stopped if it became apparent that implementation of the wait list reduction strategies was having a significant adverse impact on patients of the service.

**10 What happens when the research project ends?**

When the research project ends, the results will be reported in a variety of ways with the intention of implementing findings from this project into policy and practice for similar services across Australia. We expect that the results will be published in academic journals, shared with health professionals through conferences and workshops, and shared with government policy makers. The results of the project will be presented internally to all staff who participated at the conclusion of the project, as well as the broader team within specialist clinics at Eastern Health.

Qualitative data will be reported as themes; supporting quotes may be used, but names will be replaced with participant codes to maintain confidentiality. You will not be personally identified in any published material or presentation.

**Part 2 How is the research project being conducted?**

By signing the consent form, you consent to the research team collecting and using personal information about you for the research project. Any information obtained in connection with this research project that can identify you will remain confidential. After the data have been collected, only a number will be used to identify your information.

All data will be kept on electronic computer files accessible only to the research team. Any information kept in hard copies will be stored in a locked filing cabinet, accessible only to the researchers. Data will be stored for a minimum period of 15 years after publication of the project, after which time any records that identify you will be deleted. Your information will only be used for the purpose of this research project, and it will only be disclosed with your permission, except as required by law.

In accordance with relevant Australian privacy and other relevant laws, you have the right to request access to the information about you that is collected and stored by the research team. You also have the right to request that any information with which you disagree be corrected. Please inform the research team member named at the end of this document if you would like to access your information.

**12** **Complaints and compensation**

In the unlikely event that you suffer any distress or psychological injury because of this research project, you should contact the research team as soon as possible. You will be assisted with arranging appropriate support, if required.

**13** **Who is organising and funding the research?**

This research project is being led by Dr Katherine Harding, in collaboration with a team of researchers and clinical experts: Associate Professor Patrick Carney (Eastern Health), Professor Nicholas Taylor (Eastern Health, La Trobe University), Dr Annie Lewis (Eastern Health, La Trobe University), Professor Julie Considine (Deakin University), Associate Professor Natasha Brusco (Monash University), Professor Luke Prendergast (La Trobe University), Dr David Snowdon (Peninsula Health, La Trobe University), Dr Mitchell Sarkies (University of Sydney), and Dr Philip Choi (Eastern Health, Monash University).

The project is being funded by a National Health and Medical Research Council (NHMRC) Partnership Project grant, with contributions from Eastern Health. The Department of Health Victoria is also a partner in the research.

No member of the research team will receive a personal financial benefit from your involvement in this research project (other than their ordinary wages).

**14 Who has reviewed the research project?**

All research in Australia involving humans is reviewed by an independent group of people called a Human Research Ethics Committee (HREC). The ethical aspects of this research project have been approved by the HREC of Eastern Health.

This project will be carried out according to the *National Statement on Ethical Conduct in Human Research (2007)*. This statement has been developed to protect the interests of people who agree to participate in human research studies.

**15 Further information and who to contact**

The person you may need to contact will depend on the nature of your query. If you want any further information concerning this project or if you have any problems which may be related to your involvement in the project, you can contact any of the following people:

*Contact details for two members of the investigator team provided.*

If you have any complaints about any aspect of the project, the way it is being conducted or any questions about being a research participant in general, then you may contact:

| Reviewing HREC name | Eastern Health Human Research Ethics Committee |
| --- | --- |
| Telephone | 03 9895 3398 |
| Email | [ethics@easternhealth.org.au](mailto:ethics@easternhealth.org.au?subject=Office%20of%20Research%20and%20Ethics%20enquiry) |

| **Title** | **WaitLESS: Applying data-driven, evidence-based**  **principles to reduce waiting lists in specialist clinics** |
| --- | --- |
| **Short Title** | **WaitLESS** |
| **Protocol Number** | 2 (04/12/2024) |
| **Project Sponsor** | La Trobe University |
| **Associate Investigators** | Ass Professor Patrick Carney (Eastern Health, Monash University)  Professor Nicholas Taylor (Eastern Health, La Trobe University)  Dr Annie Lewis (Eastern Health, La Trobe University)  Professor Julie Considine (Deakin University)  Ass Professor Natasha Brusco (Monash University)  Professor Luke Prendergast (La Trobe University)  Dr David Snowdon (Peninsula Health, La Trobe University)  Dr Mitchell Sarkies (University of Sydney)  Dr Philip Choi (Eastern Health, Monash University) |
| **Location** | Eastern Health |

**Consent Form - Eastern Health staff participants – focus groups**

**Declaration by Participant**

- I have read the Participant Information Sheet.
- I understand the purposes, procedures and risks of the research described in the project.
- I have had an opportunity to ask questions, and I am satisfied with the answers received.
- I freely agree to participate in this research project as described and understand that I am free to withdraw at any time during the project without affecting my future care.
- I understand that I will be given a signed copy of this document to keep.

| Name of Participant (please print): | |
| --- | --- |
| Signature: | Date: |

**Declaration by Researcher^†^**

I have given a verbal explanation of the research project, its procedures and risks, and I believe that the participant has understood that explanation.

| Name of Researcher (please print): | |
| --- | --- |
| Signature: | Date: |

^†^ An appropriately qualified member of the research team must provide the explanation of, and information concerning, the research project.

*Note: All parties signing the consent section must date their own signature.*

**Withdrawal of Consent Form - Eastern Health staff participants – focus groups**

| **Title** | **WaitLESS: Applying data-driven, evidence-based**  **principles to reduce waiting lists in specialist clinics** |
| --- | --- |
| **Short Title** | **WaitLESS** |
| **Protocol Number** | 2 (04/12/2024) |
| **Project Sponsor** | La Trobe University |
| **Associate Investigators** | Ass Professor Patrick Carney (Eastern Health, Monash University)  Professor Nicholas Taylor (Eastern Health, La Trobe University)  Dr Annie Lewis (Eastern Health, La Trobe University)  Professor Julie Considine (Deakin University)  Ass Professor Natasha Brusco (Monash University)  Professor Luke Prendergast (La Trobe University)  Dr David Snowdon (Peninsula Health, La Trobe University)  Dr Mitchell Sarkies (University of Sydney)  Dr Philip Choi (Eastern Health, Monash University) |
| **Location** | Eastern Health |

**Declaration by Participant**

I wish to withdraw from participation in the above research project and understand that such withdrawal will not affect my employment, or my relationships with the researchers or Eastern Health.

| Name of Participant (please print): | |
| --- | --- |
| Signature: | Date: |

In the event that the participant’s decision to withdraw is communicated verbally, the Senior Researcher must provide a description of the circumstances below.

|  |
| --- |

**Declaration by Researcher^†^**

I have given a verbal explanation of the implications of withdrawal from the research project, and I believe that the participant has understood that explanation.

| Name of Researcher (please print): | |
| --- | --- |
| Signature: | Date: |

^†^ An appropriately qualified member of the research team must provide information concerning withdrawal from the research project.

*Note: All parties signing the consent section must date their own signature.*

**Participant Information Sheet and Consent Form**

**Consumer interviews**

| **Title** | **WaitLESS: Applying data-driven, evidence-based**  **principles to reduce waiting lists in specialist clinics** |
| --- | --- |
| **Short Title** | **WaitLESS** |
| **Protocol Number** | 2 (04/12/2024) |
| **Project Sponsor** | La Trobe University |
| **Associate Investigators** | Ass Professor Patrick Carney (Eastern Health, Monash University)  Professor Nicholas Taylor (Eastern Health, La Trobe University)  Dr Annie Lewis (Eastern Health, La Trobe University)  Professor Julie Considine (Deakin University)  Ass Professor Natasha Brusco (Monash University)  Professor Luke Prendergast (La Trobe University)  Dr David Snowdon (Peninsula Health, La Trobe University)  Dr Mitchell Sarkies (University of Sydney)  Dr Philip Choi (Eastern Health, Monash University) |
| **Location** | Eastern Health |

**Part 1**  **What does my participation involve?**

1. **Introduction**

You are invited to take part in this research project, which is called *“****WaitLESS: Applying data-driven, evidence-based*** ***principles to reduce waiting lists in specialist clinics.”*** You have been invited because you attended an appointment with a clinic involved in this project and completed an expression of interest to participate.

This Participant Information Sheet and Consent Form tells you about the research project. It explains the processes involved with taking part. Knowing what is involved will help you decide if you want to take part in the research.

Please read this information carefully. Ask questions about anything that you don’t understand or want to know more about. Before deciding whether or not to take part, you might want to talk about it with a relative, friend or local health worker.

Participation in this research is voluntary. If you don’t wish to take part, you don’t have to.

If you decide you want to take part in the research project, you will be asked to sign the consent section. By signing it you are telling us that you:

• Understand what you have read

• Consent to take part in the research project

• Consent to be involved in the research described

• Consent to the use of your personal and health information as described.

You will be given a copy of this Participant Information and Consent Form to keep.

**2**  **What is the purpose of this research?**

Wait lists are a widespread problem in health services. The WaitLESS project aims to demonstrate how waiting lists for outpatient specialist appointments can be reduced using practical, cost-effective strategies that are not reliant on additional medical specialists.

This qualitative study forms one aspect of the WaitLESS project and is intended to find out about the experience of patients who attend the specialist clinics involved in the trial. We will interview patients who attend the services during the periods before and after changes are made to each clinic to see if their experiences differ. We also want to learn about what is done well in the clinics and what could be done to improve the experience for patients.

**3** **What does participation in this research involve?**

If you agree to participate, you will be invited to participate in an interview to discuss your experience with the specialist medical clinic. We want to find out your experience and perceptions of the clinic service, throughout the journey from referral and booking, attendance at the first appointment, and follow up.

The interview will be conducted either in person at a location convenient for you (such as an Eastern Health site or your home) or by video-conference (according to your preference) and take between 30-45 minutes. It will be conducted by a member of our research team.

This research project has been designed to make sure the researchers interpret the results in a fair and appropriate way and avoids researchers or participants jumping to conclusions. The interview will be audio recorded. After the interview, we will transcribe the audio recording and then delete the recording. We will send you a copy of the transcript from your interview, and you will have an opportunity to check the transcript, and to add additional thoughts on the topic that you would like to contribute.

There are no costs associated with participating in this research project. You will be offered a $50 gift card as a token of appreciation for your time.

**4** **Other relevant information about the research project**

The primary aim of this study is to determine whether the WaitLESS strategies are effective in reducing waiting lists for specialist clinics. To do this, data relating to waiting times will be collected before and after implementation of waiting list reduction strategies. We are also collecting a range of other information about service, patient, process, and cost outcomes.

Other patients will also be invited to participate in an interview, at different time points in the project. The interview data will assist us to understand the impact of the project on specialist medical clinics, from the perspective of the people who use the services.

**5** **Do I have to take part in this research project?**

Participation in any research project is voluntary. If you do not wish to take part, you do not have to. If you decide to take part and later change your mind, you are free to withdraw from the project at any stage.

Your decision whether to take part or not to take part, or to take part and then withdraw, will not affect your routine care, your relationship with professional staff, or your relationship with Eastern Health.

**6** **What are the possible benefits of taking part?**

There will be no clear benefit to you from your participation in this research. However, we hope to learn whether the WaitLESS project strategies will reduce wait times in specialist clinics at Eastern Health and whether the findings could be further generalised. If successful, the project may lead to direct benefits for future patients at this, and other, health services.

**7** **What are the possible risks and disadvantages of taking part?**

There are no foreseeable risks to you in taking part in this project. In the unlikely event that you find any of the interview questions stressful or upsetting, you may stop immediately. If you become upset or distressed because of your participation in the research project, the research team can assist with arranging appropriate support.

**8** **What if I withdraw from this research project?**

If you do consent to participate, you may withdraw at any time. If you decide to withdraw from the project, please notify the research officer. You will be provided with and asked to complete and sign a ‘Withdrawal of Consent’ form.

If you decide to leave the research project, the researchers will not collect additional personal information from you. You should be aware that data collected up to the time you withdraw will form part of the research project results. If you do not want your data to be included, you must tell the researchers when you withdraw from the research project.

**9** **Could this research project be stopped unexpectedly?**

It is possible, but unlikely, that this research project will be stopped unexpectedly. For example, the project may be stopped if it became apparent that implementation of the wait list reduction strategies was having a significant adverse impact on patients of the service.

**10** **What happens when the research project ends?**

When the research project ends, the results will be reported in a variety of ways with the intention of implementing findings from this project into policy and practice for similar services across Australia. We expect that the results will be published in academic journals, shared with health professionals through conferences and workshops, and shared with government policy makers. Only group data will be reported. You will not be able to be personally identified in any published material. If you are interested, a summary of the results of the project will be sent to you at the conclusion of the project. Please inform the researcher who conducts your interview if you would like to receive a summary of project results.

**Part 2** **How is the research project being conducted?**

By signing the consent form, you consent to the research team collecting and using personal information about you for the research project. Any information obtained in connection with this research project that can identify you will remain confidential. After the data have been collected, only a number will be used to identify your information.

All data will be kept on electronic computer files accessible only to the research team. Any information kept in hard copies will be stored in a locked filing cabinet, accessible only to the researchers. Data will be stored for a period of 15 years after the conclusion of the project, after which time it will be deleted. Your information will only be used for the purpose of this research project, and it will only be disclosed with your permission, except as required by law.

In accordance with relevant Australian privacy and other relevant laws, you have the right to request access to the information about you that is collected and stored by the research team. You also have the right to request that any information with which you disagree be corrected. Please inform the research team member named at the end of this document if you would like to access your information.

**12** **Complaints and compensation**

In the unlikely event that you suffer any distress or psychological injury as a result of this research project, you should contact the research team as soon as possible. You will be assisted with arranging appropriate support, if required.

**13** **Who is organising and funding the research?**

This research project is being led by Professor Katherine Harding, in collaboration with a team of researchers and clinical experts: Associate Professor Patrick Carney (Eastern Health, Monash University), Professor Nicholas Taylor (Eastern Health, La Trobe University), Dr Annie Lewis (Eastern Health, La Trobe University), Professor Julie Considine (Deakin University), Associate Professor Natasha Brusco (Monash University), Professor Luke Prendergast (La Trobe University), Dr David Snowdon (Peninsula Health, La Trobe University), Dr Mitchell Sarkies (University of Sydney), and Dr Philip Choi (Eastern Health, Monash University).

The project is being funded by a National Health and Medical Research Council (NHMRC) Partnership Project grant.

No member of the research team will receive a personal financial benefit from your involvement in this research project (other than their ordinary wages).

**14** **Who has reviewed the research project?**

All research in Australia involving humans is reviewed by an independent group of people called a Human Research Ethics Committee (HREC). The ethical aspects of this research project have been approved by the HREC of Eastern Health.

This project will be carried out according to the *National Statement on Ethical Conduct in Human Research (2007)*. This statement has been developed to protect the interests of people who agree to participate in human research studies.

**15** **Further information and who to contact**

The person you may need to contact will depend on the nature of your query.

If you want any further information concerning this project or if you have any problems which may be related to your involvement in the project, you can contact:

*Contact details for two members of the investigator team provided.*

If you have any complaints about any aspect of the project, the way it is being conducted or any questions about being a research participant in general, then you may contact:

| Reviewing HREC name | Eastern Health Human Research Ethics Committee |
| --- | --- |
| Telephone | 03 9895 3398 |
| Email | [ethics@easternhealth.org.au](mailto:ethics@easternhealth.org.au?subject=Office%20of%20Research%20and%20Ethics%20enquiry) |

**Consent Form**

| **Title** | **WaitLESS: Applying data-driven, evidence-based**  **principles to reduce waiting lists in specialist clinics** |
| --- | --- |
| **Short Title** | **WaitLESS** |
| **Protocol Number** | 2 (04/12/2024) |
| **Project Sponsor** | La Trobe University |
| **Associate Investigators** | Ass Professor Patrick Carney (Eastern Health, Monash University)  Professor Nicholas Taylor (Eastern Health, La Trobe University)  Dr Annie Lewis (Eastern Health, La Trobe University)  Professor Julie Considine (Deakin University)  Ass Professor Natasha Brusco (Monash University)  Professor Luke Prendergast (La Trobe University)  Dr David Snowdon (Peninsula Health, La Trobe University)  Dr Mitchell Sarkies (University of Sydney)  Dr Philip Choi (Eastern Health, Monash University) |
| **Location** | Eastern Health |

**Declaration by Participant**

I have read the Participant Information Sheet or someone has read it to me in a language that I understand.

I understand the purposes, procedures and risks of the research described in the project.

I have had an opportunity to ask questions and I am satisfied with the answers I have received.

I freely agree to participate in this research project as described and understand that I am free to withdraw at any time during the project without affecting my future care.

I understand that I will be given a signed copy of this document to keep.

| Name of Participant (please print): | |
| --- | --- |
| Signature: | Date: |

**Declaration by Researcher^†^**

I have given a verbal explanation of the research project, its procedures and risks, and I believe that the participant has understood that explanation.

| Name of Participant (please print): | |
| --- | --- |
| Signature: | Date: |

^†^ An appropriately qualified member of the research team must provide the explanation of, and information concerning, the research project.

Note: All parties signing the consent section must date their own signature.

**Form for Withdrawal of Participation -** *Adult providing own consent*

| **Title** | **WaitLESS: Applying data-driven, evidence-based**  **principles to reduce waiting lists in specialist clinics** |
| --- | --- |
| **Short Title** | **WaitLESS** |
| **Protocol Number** | 2 (04/12/2024) |
| **Project Sponsor** | La Trobe University |
| **Associate Investigators** | Ass Professor Patrick Carney (Eastern Health, Monash University)  Professor Nicholas Taylor (Eastern Health, La Trobe University)  Dr Annie Lewis (Eastern Health, La Trobe University)  Professor Julie Considine (Deakin University)  Ass Professor Natasha Brusco (Monash University)  Professor Luke Prendergast (La Trobe University)  Dr David Snowdon (Peninsula Health, La Trobe University)  Dr Mitchell Sarkies (University of Sydney)  Dr Philip Choi (Eastern Health, Monash University) |
| **Location** | Eastern Health |

**Declaration by Participant**

I wish to withdraw from participation in the above research project and understand that such withdrawal will not affect my routine care, or my relationships with the researchers or Eastern Health.

| Name of Participant (please print): | |
| --- | --- |
| Signature: | Date: |

In the event that the participant’s decision to withdraw is communicated verbally, the Senior Researcher must provide a description of the circumstances below.

|  |
| --- |

**Declaration by Researcher^†^**

I have given a verbal explanation of the implications of withdrawal from the research project and I believe that the participant has understood that explanation.

| Name of Participant (please print): | |
| --- | --- |
| Signature: | Date: |

^†^ An appropriately qualified member of the research team must provide information concerning withdrawal from the research project.

Note: All parties signing the consent section must date their own signature.
