# Supplementary material for: Application of the STAT model for demand management to reduce specialist clinic waiting times: protocol for the WaitLESS stepped wedge cluster randomised controlled trial
Source: BMJ Open. 2026 Jul 23;16(7):e115253. doi: 10.1136/bmjopen-2025-115253 (PMC13404491; doi:10.1136/bmjopen-2025-115253)
Supplement: online supplemental file 3 [file bmjopen-16-7-s003.pdf]

Eastern Health

Office of Research and Ethics
Eastern Health
Level 2, 5 Arnold Street
Box Hill VIC 3128

T: 03 9895 3398
F: 03 9094 9610
E: ethics@easternhealth.org.au
www.easternhealth.org.au
ABN 68 223 819 017

9 January 2025

Prof Katherine Harding
Allied Health Clinical Research Office
5 Arnold St
Box Hill

Dear Katherine

Study Title: WaitLESS: Waiting List Evidence to Support Specialist clinics

---

Principal Investigator: Katherine Harding
Associate Investigator/s: Patrick Carney, Nicholas Taylor, Annie Lewis, Julie Considine, Natasha Brusco, David Snowden, Luke Prendegast, Mitchell Sarkies, Phillip Choi
Study Coordinator/s: Nil
Eastern Health Reference Number: E24-020-11382

Thank you for your correspondence on 10Dec2024 addressing the matters raised in the HREC's letter dated 25Dec2024 following the ethical review of the above project at its meeting held out of session. Final correspondence was received on 07Jan2025 including series of amendments and clarifications.

I am pleased to advise that the above study has received full ethical approval from the Eastern Health Human Research Ethics Committee (HREC). The study has received full governance authorisation also from the Office of the Chief Executive Officer or delegate at Eastern Health and may commence at the nominated site.

The HREC confirms that your proposal meets the requirements of the National Statement on Ethical Conduct in Human Research (2023) (**National Statement**). This HREC is organised and operates in accordance with the National Health and Medical Research Council's (**NHRMC**) National Statement and all subsequent updates, and in accordance with the Note for Guidance on Good Clinical Practice (CPMP/ICH/135/95), the Health Privacy Principles (HPP's) described in the Health Records Act 2001 (Vic).

HREC Final Approval Date: 07 Jan 2024
Research Governance Authorisation: 07 Jan 2024
Ethical approval for this project applies at the following site/s: Eastern Health – Care at Home and Acute Specialist Clinics

Approved Documents:

The following documents have been reviewed and **approved** by the HREC:

Document	Version	Dated
Human Research Ethics Application (HREA) – ERM Review Reference: HREC/111382/EH-2024-460539(v2)	2	12 Dec 2024
Protocol (clean and tracked)	2	4 Dec 2024
Participant Information Sheet and Consent Form (PICF) – Staff Focus Groups	2	4 Dec 2024
Participant Information Sheet and Consent Form (PICF) – Consumer Interviews	2	4 Dec 2024
Staff Demographic questionnaire (Clean and Tracked)	2	4 Dec 2024
WaitLESS Staff Demographic Questionnaire	2	04 Dec 2024
WaitLess Consumer Participant Interview Guide	2	04 Dec 2024
WaitLESS patient Flyer	2	4 Dec 2024

The following documents have been reviewed and **noted** by the HREC:

Document	Version	Dated
Victorian Specific Module (VSM)	1	07 Jan 2025
WaitLESS Staff training schedule and resources	2	4 Dec 2024
WaitLESS data capture template	2	4 Dec 2024

The following documents have been reviewed and authorised / noted by the Office of Research and Ethics (Research Governance):

Document	Version	Dated
Site Specific Assessment (SSA) – ERM	2	07 Jan 2024
NHMRC Partnership Grant (GNT2035258)	N/A	05 Jan 2024

Approval is subject to:

1. The Principal Researcher is to ensure that all associate researchers are aware of the terms of approval and to ensure the project is conducted as specified in the application and in accordance with the National Statement.
2. Immediate notification to the Office of Research & Ethics of any serious adverse events on participants.
3. Immediate notification of any unforeseen events that may affect the continuing ethical acceptability of the project.
4. Immediate notification to the Office of Research and Ethics of any breach of data, breach of participant's rights and participant complaints.
5. Notification of any changes to personnel on the study.
6. Notification and reasons for ceasing the project prior to its expected date of completion.

7. Submission to the HREC of any proposed modifications to the project or documents as approved by the HREC and noted above.
8. Submission of an **annual report due in February each year** for the preceding calendar year, for the duration of the study. Further guidance can be found https://www.easternhealth.org.au/research-ethics/guidance
9. Submission of a **final report** and papers published on completion of the project.
10. Projects may be subject to an audit or any other form of monitoring by the Eastern Health Office of Research & Ethics at any time.

Research Governance Authorisation
Research governance/site authorisation is required at all sites participating in the study. The study must be authorised at a site before the research project can commence.
A copy of this ethics approval letter and all relevant documents must be submitted to site for authorisation by the Chief Executive or delegate. This applies to each site participating in the research.

Confidentiality, Privacy & Research:
Research data stored on personal computers, USBs and other portable electronic devices must not be identifiable. No patients' names or UR numbers must be stored on these devices. Electronic storage devices must be password protected or encrypted. The conduct of research must be compliant with the conditions of ethics approval and Eastern Health policies.

Publications:
Publications are very important for disseminating research and demonstrating the research activity of investigators and their institutions. Publications provide evidence of the contribution that participants, researchers and funding sources make. It is therefore important that the role of Eastern Health including the investigator's Eastern Health affiliation (if appropriate) is provided in publications.

Composition of the HREC
We confirm at the meetings at which the above project was considered, the Committee fulfilled the requirements of the NHMRC National Statement in that it contained men and women encompassing different age groups and included people in the following categories:

HREC Members
Chairperson
Person/s bringing a broader community or consumer perspective
Lawyer
Person/s fulfilling a Pastoral Care Role
Person/s with knowledge of and current experience in the professional care or treatment of people
Person/s with Current Research Experience

Please always quote the Eastern Health Reference Number **E24-020-111382** in all future correspondence.

Yours sincerely

[redacted]

09 Jan 2024

[redacted]
Ethics Governance and Project Officer
Eastern Health Office of Research and Ethics

On behalf of

1. Eastern Health Human Research Ethics Committee (Ethics Approval)
2. Director of Eastern Health Institute (Site Research Governance Authorisation)
