# Supplementary material for: Application of the STAT model for demand management to reduce specialist clinic waiting times: protocol for the WaitLESS stepped wedge cluster randomised controlled trial
Source: BMJ Open. 2026 Jul 23;16(7):e115253. doi: 10.1136/bmjopen-2025-115253 (PMC13404491; doi:10.1136/bmjopen-2025-115253)
Supplement: Supplementary file 4 [file bmjopen-2025-115253.reviewer_comments.pdf]

PEER REVIEW HISTORY

BMJ Open publishes all reviews undertaken for accepted manuscripts. Reviewers are asked to complete a checklist review form and are provided with free text boxes to elaborate on their assessment. These free text comments are reproduced below.

ARTICLE DETAILS

Title (Provisional)

Application of the STAT model for demand management to reduce specialist clinic waiting times: Protocol for the WaitLESS stepped wedge cluster randomised controlled trial

Authors

Harding, Katherine E.; Carney, Patrick; Lewis, Annie K; Considine, Julie; Brusco, Natasha; Prendergast, Luke; Snowdon, David; Sarkies, Mitchell N; Choi, Philip; Bould, Em; Taylor, Nicholas F

VERSION 1 - REVIEW

**Reviewer**	**1**
**Name**	**Reyes-Santías, Francisco**
**Affiliation**	**Universidad de Vigo, Organización de Empresas e Mercadotecnia**
**Date**	**15-Apr-2026**
**COI**	**None**

Critical Analysis: "APPLICATION OF THE STAT MODEL FOR DEMAND MANAGEMENT TO REDUCE SPECIALIST CLINIC WAITING TIMES: PROTOCOL FOR THE WAITLESS STEPPED WEDGE CLUSTER RANDOMISED CONTROLLED TRIAL"

Journal: *BMJ Open*

1. Strengths & Methodological Rigour

The protocol demonstrates several notable strengths. First, the use of a **stepped wedge cluster randomised controlled trial (RCT)** design is appropriate for a health service intervention where simultaneous rollout across all sites is impractical or unethical, and where the intervention is expected to do more good than harm. This design enhances internal validity compared to pre-post studies.

Second, the **hybrid type II implementation-effectiveness design** is a major asset. By simultaneously measuring clinical effectiveness (waiting times) and implementation outcomes (fidelity, acceptability, cost), the study acknowledges that even an efficacious model will fail if not adopted or sustained in real-world settings. This dual focus increases the translational relevance of the findings.

Third, the **five-step STAT implementation process** is clearly operationalised, moving

from demand analysis to backlog reduction and capacity protection. This logical sequence addresses both legacy waiting lists and future demand—a key differentiator from simpler triage models.

Finally, the inclusion of **cost-effectiveness analysis from a health service perspective** is critical. Without this, even successful reductions in wait times may be dismissed by budget-holding decision-makers if implementation costs are prohibitive.

2. Methodological Limitations & Concerns

Despite its strengths, several limitations warrant critique:

- **Unit of analysis and contamination risk:** Clusters are defined as "eight clinical specialties." However, within a single metropolitan health network, patient referrals, staffing, and administrative processes may overlap. The stepped wedge design assumes that once a cluster crosses over, it remains unidirectionally exposed. But if surgeons or physicians work across multiple specialty clinics (e.g., both general medicine and subspecialty cardiology), contamination could bias effect estimates toward the null.
- **Primary outcome definition:** The primary effectiveness outcome is "waiting time from referral to first appointment." This is a process measure, not a clinical outcome. Longer waiting times are only problematic insofar as they worsen health outcomes, morbidity, or patient-reported experiences. The protocol does not specify whether the trial will measure clinical deterioration, avoidable emergency presentations, or patient quality of life—leaving a gap in the evidence that waiting time reduction actually improves patient health.
-
- **No mention of blinding or independent outcome assessment.** Given the collaborative implementation between researchers and clinical teams, knowledge of intervention timing could influence behaviour (e.g., Hawthorne effect) or data recording (e.g., more aggressive list cleaning post-intervention). The paper lacks detail on how outcome assessors will be blinded or how data integrity will be safeguarded.
- **Generalisability constraints:** The trial is confined to a single metropolitan health network in Melbourne, Australia. Healthcare systems in rural, remote, or non-Australian contexts (e.g., low- and middle-income countries, privatised or insurance-based systems) face different demand-supply dynamics, staffing models, and referral pathways. The STAT model's transportability beyond this setting is unknown.

3. Implementation & Practical Considerations

The protocol's six-month pre-implementation period and three-monthly stepped crossovers are ambitious. However, the paper does not address how the study will manage **external shocks** (e.g., pandemic surges, industrial action, seasonal respiratory illnesses) that could independently alter waiting times. Linear mixed effects models can adjust for time, but unmeasured time-varying confounders (e.g., sudden staff departures, IT system failures) remain a threat.

Furthermore, the implementation strategies are described as "collaboratively implemented by researchers and clinical teams." This raises questions about **sustainability post-trial**. If researchers drive the process, fidelity and adoption may

decline once external support is withdrawn. The protocol would benefit from explicit measurement of sustainment capacity.

4. Missing Ethical & Equity Considerations

The paper does not mention how the study will address potential **unintended consequences** of demand management. For example, prioritising patients via the STAT model could systematically disadvantage certain groups (e.g., those with limited digital literacy, non-English speakers, or patients with complex multimorbidity who require longer appointments). Without equity-stratified analyses, the intervention could reduce average waiting times while worsening disparities.

5. Conclusion

The WaitLESS trial protocol represents a well-designed, methodologically sophisticated effort to translate demand-driven strategies into specialist outpatient care. The hybrid stepped wedge design and inclusion of implementation and cost outcomes are commendable. However, the absence of clinical outcome measures, potential contamination risks, unclear blinding procedures, and limited generalisability temper enthusiasm. If the trial demonstrates effectiveness on waiting times, future research must confirm whether this translates to better health outcomes, reduced inequities, and sustained adoption without research support. Publish with Minor Revisions.

VERSION 1 - AUTHOR RESPONSE

WaitLESS Protocol – Response to reviewers

Comment

The protocol demonstrates several notable strengths. First, the use of a stepped wedge cluster randomised controlled trial (RCT) design is appropriate for a health service intervention where simultaneous rollout across all sites is impractical or unethical, and where the intervention is expected to do more good than harm. This design enhances internal validity compared to pre-post studies.
Second, the hybrid type II implementation-effectiveness design is a major asset. By simultaneously measuring clinical effectiveness (waiting times) and implementation outcomes (fidelity, acceptability, cost), the study acknowledges that even an efficacious model will fail if not adopted or sustained in real-world settings. This dual focus increases the translational relevance of the findings.
Third, the five-step STAT implementation process is clearly operationalised, moving from demand analysis to backlog reduction and capacity protection. This logical sequence addresses both legacy waiting lists and future demand—a key differentiator from simpler triage models.
Finally, the inclusion of cost-effectiveness analysis from a health service perspective is critical. Without this, even successful reductions in wait times may be dismissed by budget-holding decision-makers if implementation costs are prohibitive.

Response

Thank you for these comments.

Comment

Unit of analysis and contamination risk: Clusters are defined as "eight clinical specialties." However, within a single metropolitan health network, patient referrals, staffing, and administrative processes may overlap. The stepped wedge design assumes that once a cluster crosses over, it remains unidirectionally exposed. But if surgeons or physicians work

across multiple specialty clinics (e.g., both general medicine and subspecialty cardiology), contamination could bias effect estimates toward the null.

Response

This is an important point for consideration and we agree that there is some risk of contamination between clusters. This is one of the realities of health services research of this nature, where interventions are implemented in large organisations and it is not possible to control all aspects of the intervention environment. This risk is being mitigated by (1) including a diverse range of clinical specialties which minimise the likelihood of the same doctors working across more than one cluster; and (2) having a structured implementation process to guide the nature and timing of changes to clinic operations. The following sentence has been added to the Design section (page 6) to acknowledge this issue:

> *While the design carries some risk of contamination due to the possibility of some of the same staff working across different clusters, this will be minimised through inclusion of a diverse range of clinical specialties and structured implementation process.*

This issue will also be noted as a limitation of the trial when results are reported.

Comment

Primary outcome definition: The primary effectiveness outcome is "waiting time from referral to first appointment." This is a process measure, not a clinical outcome. Longer waiting times are only problematic insofar as they worsen health outcomes, morbidity, or patient-reported experiences. The protocol does not specify whether the trial will measure clinical deterioration, avoidable emergency presentations, or patient quality of life—leaving a gap in the evidence that waiting time reduction actually improves patient health.

Response

We agree that waiting time is different from clinical outcomes, but this doesn't make it less valid as a primary outcome for the trial. The STAT model aims to reduce waiting time, and this is justified as a worthwhile endeavour give that there is an expansive body of evidence that links longer wait times to worsening clinical outcomes, as well as secondary impacts on quality of life related to stress, anxiety, reduced community participation and increased use of other services during the waiting period. Patient-reported experiences are being captured through qualitative interviews with a cohort of patients attending the clinics before and after the intervention is introduced, and emergency department presentations are also being captured for cohorts of patients in the periods before and after their index appointment in the pre and post intervention phases at each participating clinic.

While more specific patient level outcomes would be of value, this is beyond the scope of the current trial, and is not something that we are able to change given that the existing protocol has been funded by a national grant and the protocol has been prospectively registered. However, we will include this as an area for further research when we report the findings.

We have added an additional sentence to the introduction to emphasise that the link between long waiting times is well-established. This adds strength to the justification of our primary aim to reduce waiting time, rather than to demonstrate that reduced waiting time improves outcomes.

Introduction, paragraph 3, now reads:

The link between delays in access to care and poorer health outcomes have been well-established for a wide range of condition.(7, 18-21) Furthermore, patients on outpatient waiting lists feel 'forgotten', are unable to plan for the future, experience deterioration in their health, are angry with the system, and express a fear of 'dying on the waiting list'.(5)

Comment
No mention of blinding or independent outcome assessment. Given the collaborative implementation between researchers and clinical teams, knowledge of intervention timing could influence behaviour (e.g., Hawthorne effect) or data recording (e.g., more aggressive list cleaning post-intervention). The paper lacks detail on how outcome assessors will be blinded or how data integrity will be safeguarded.

Response

Primary outcome data is from health service reports generated independently by the centralised data unit at the health service. The process to obtain these data involves requests to the data unit, and the staff generating these data from hospital records are independent from the research team. The primary outcome (waiting time) is calculated as the time lapse between the date on referrals received and the date of first attendance, which cannot be realistically manipulated, intentionally or unintentionally, by people involved in the project.

The independence of the staff providing primary outcome data is noted on page 9, under Randomisation and masking:

Clinic patients will not be aware of the study, and routinely collected data contributing to the primary outcome measure (referral and appointment dates used to calculate waiting time, patients allocated to the waiting list) are collected by a centralised data management service within the health organisation independent from the research team.

Comment

Generalisability constraints: The trial is confined to a single metropolitan health network in Melbourne, Australia. Healthcare systems in rural, remote, or non-Australian contexts (e.g., low- and middle-income countries, privatised or insurance-based systems) face different demand-supply dynamics, staffing models, and referral pathways. The STAT model's transportability beyond this setting is unknown.

Response

We agree that this is a limitation of the trial, and will note this when findings are reported. BMJ Open does not allow a discussion session in protocols, but we have added a sentence to better justify the use of a single health service in the "Setting" section (end of paragraph 1 under setting on page 6) to acknowledge this concern:

The inclusion of a single health service could be seen as a limitation, but the size of the health network and its operation across multiple sites spanning a diverse geographical catchment provides confidence in the applicability of the findings to a broad range of health settings.

Comment

The protocol's six-month pre-implementation period and three-monthly stepped crossovers are ambitious. However, the paper does not address how the study will manage external shocks (e.g., pandemic surges, industrial action, seasonal respiratory illnesses) that could independently alter waiting times. Linear mixed effects models can adjust for time, but unmeasured time-varying confounders (e.g., sudden staff departures, IT system failures) remain a threat.
Furthermore, the implementation strategies are described as "collaboratively implemented by researchers and clinical teams." This raises questions about sustainability post-trial. If researchers drive the process, fidelity and adoption may decline once external support is withdrawn. The protocol would benefit from explicit measurement of sustainment capacity.

Response

We agree that accounting for disruptions that are outside of the control of the trial is a significant challenge in stepped wedge trial designs. Some contingencies for this are already place, but in response to this comment we have made some minor changes to strengthen this aspect of our analysis plan. If such shocks should occur that are not sufficiently accounted for in the existing modelling, we have added the option to include a disruption indicator variable for the disrupted time period to adjust for abrupt deviations. This model adjustment to disruption can also be included as an indicator within affected clusters only. While a disruption indicator can be time-varying, this approach is still valid under the assumption that disruptions are external intercurrent events and not induced by intervention. We also have capacity to use time series data for additional sensitivity analyses and have explicitly added the option to include within-cluster time-series sensitivity analyses into the protocol to account for localised temporal disruptions while assessing the persistence of intervention effects.

The analysis section has been modified to include these elements, and now reads as follows (Analyses section, Effectiveness, final paragraph (page 15):

> *Linear mixed effects models will be used to analyse data related to the primary outcome with time-point (time since trial start) and time since intervention as factor variables. This allows for the expected nonlinear change over time, as well as an analysis of persistence of intervention and time-averaged intervention effects following the service redesign, consistent with recommendations.(40) Generalised linear mixed effects models will be used to analyse the secondary outcomes (binomial for proportions and negative binomial for counts to allow for over-dispersion). A random effect for clinic will be used for all models to account for clustering. Visual diagnostics will be used to assess model assumption validity and transformations of outcomes or robust estimators will be used as needed. Adjusting for, and analysing association between, covariates (e.g. referral source and demographic data, including potential indicators of disadvantage such need for interpreters) and outcomes will also be completed by including these in the mixed effects models as predictors. In the event of any system-wide (e.g. pandemic) and within-cluster notable disruptions (e.g. staffing issues) that are not adequately captured by the baseline random-effects structure and residual variation, a disruption indicator variable will be used for the disrupted time period. This model of adjustment to disruption can also be included as an indicator within affected clusters only. As an additional sensitivity analysis, within-cluster time-series analyses will be conducted to account for localised temporal disruptions while assessing the persistence of intervention effects. R version 4.4.1 (or newer version if available) will be used for the analyses.*

We agree that sustainability post-trial is a very important consideration but is beyond the scope of the current trial and available funding. This will be acknowledged as an area for further research when the findings are published. A minor amendment has been made to the introduction to note that we already have some evidence that outcomes can be sustained (p 5):

> *Early success in a single pilot study in a single specialist outpatient clinic that almost eliminated a waiting list of 600 patients and sustained the service with minimal waiting lists over 4 years suggests it is feasible to apply the STAT model to specialist clinic settings, but further research is required to see whether it can be implemented at scale (27, 28).*

Comment

The paper does not mention how the study will address potential unintended consequences of demand management. For example, prioritising patients via the STAT model could systematically disadvantage certain groups (e.g., those with limited digital literacy, non-English speakers, or patients with complex multimorbidity who require longer appointments). Without equity-stratified analyses, the intervention could reduce average waiting times while worsening disparities.

Response

We agree that this is an important consideration. Covariates such as demographic data and referral source will be included as variables in the linear mixed effects models, and this demographic data will include some indicators of potential disadvantage such as need for an interpreter belonging to a first nations community. In addition, our staff focus groups will include discussion about changes that clinicians observe from their experience, which should highlight any increasing challenges in managing patients requiring longer appointments, for example. Finally, our protocol also includes qualitative interviews with patients that may highlight personal stories of both improved experience as well as unintended consequences. These measures will provide some indication of any change to patterns in management of disadvantaged groups, although we will also acknowledge that this will be an area requiring further investigation in the future.

To better acknowledge the specific inclusion of variables related to disadvantage in the linear fixed effect models, we have made a small adjustment to the wording under the "effectiveness" section of the Analysis section on page 15:

> *Adjusting for, and analysing association between, covariates (e.g. referral source and demographic data, including potential indicators of disadvantage such as need for interpreters) and outcomes will also be completed by including these in the mixed effects models as predictors. R version 4.4.1 (or newer version if available) will be used for the analyses.*

Comment

The WaitLESS trial protocol represents a well-designed, methodologically sophisticated effort to translate demand-driven strategies into specialist outpatient care. The hybrid stepped wedge design and inclusion of implementation and cost outcomes are commendable. However, the absence of clinical outcome measures, potential contamination risks, unclear blinding procedures, and limited generalisability temper enthusiasm. If the trial demonstrates effectiveness on waiting times, future research must confirm whether this translates to better health outcomes, reduced inequities, and sustained adoption without research support.

Response

Thank you. We hope that the changes made in response to your comments help to alleviate some of these concerns. We wholeheartedly agree that if the trial demonstrates that the STAT model is successful in reducing waiting times in specialist clinics, translation to health outcomes, reduced inequities and sustained adoption without research support will be priorities for future research.

VERSION 2 - REVIEW

**Reviewer**	**1**
**Name**	**Reyes-Santías, Francisco**
**Affiliation**	**Universidad de Vigo, Organización de Empresas e Mercadotecnia**
**Date**	**30-May-2026**
**COI**	

REVIEW OF THE PAPER: "APPLICATION OF THE STAT MODEL FOR DEMAND MANAGEMENT TO REDUCE SPECIALIST CLINIC WAITING TIMES: PROTOCOL FOR THE WaitLESS STEPPED WEDGE CLUSTER RANDOMISED CONTROLLED TRIAL"

Journal: *BMJ Open*

Overall Assessment

The proposed WaitLESS trial represents a methodologically rigorous approach to evaluating the real-world implementation of the STAT model. The use of a hybrid type II effectiveness-implementation design with a stepped wedge cluster RCT is appropriate given the practical constraints of introducing an intervention sequentially across hospital clinics. The protocol addresses a significant problem (specialist outpatient wait times) with a previously evidence-informed model.

Strengths

1. **Appropriate Study Design:** The stepped wedge cluster RCT is well-suited for health system interventions where it is logistically or ethically difficult to withhold the intervention (STAT model) from some clusters for the entire study period. Randomizing the order of implementation across eight specialties strengthens causal inference compared to a pre-post design.

2. **Hybrid Type II Design:** This is a major strength. By measuring both effectiveness (wait times) and implementation outcomes (fidelity, acceptability, cost), the study will generate actionable knowledge about *how* and *why* the STAT model works (or fails) in real-world practice, not just whether it works.

3. **Clear Primary Outcome:** The primary effectiveness outcome (waiting time from referral to first appointment) is patient-centered, clinically meaningful, and objectively measurable from healthcare data.

4. **Comprehensive Implementation Framework:** Mapping qualitative data to the Consolidated Framework for Implementation Research (CFIR) will allow for systematic identification of barriers and facilitators, enhancing generalizability to other settings.

5. **Collaborative Implementation Strategy:** Involving researchers and clinical teams together increases the likelihood of contextual adaptation and buy-in, which is critical for implementation success.

Methodological Concerns & Limitations

1. **Risk of Contamination:** In a stepped wedge design within a single metropolitan health network, there is a risk that staff from clusters not yet randomized to start the STAT model might learn about the model from colleagues in active clusters, or that system-wide changes (e.g., a new electronic referral system) could affect all clusters simultaneously. The paper does not mention how contamination will be mitigated.
2. **Statistical Power & Clustering:** The analysis plan mentions linear mixed effects models, which is correct for clustered data. However, with only eight clusters (specialties), the study may have limited power to detect a modest effect size, especially if there is high intracluster correlation (i.e., waiting times vary more between specialties than within specialties over time). The paper does not report a sample size or power calculation.

3. **Unit of Analysis Issue:** The primary analysis appears to be at the patient level (referral to appointment time), but randomization is at the cluster level. This is statistically appropriate with mixed models, but the paper does not specify how degrees of freedom for the intervention effect will be handled (e.g., using cluster-robust standard errors or Kenward-Roger approximation), which is critical with few clusters.

4. **Step Duration:** A three-month interval between cluster starts is relatively short. Given the six-month pre-implementation and six-month follow-up, it is unclear if this allows sufficient time for each specialty to complete all five STAT steps (especially step iii: address existing backlogs) before the next clusters begin. Implementation fatigue could be an issue.

5. **Missing Key Details on Cost-Effectiveness:** The paper states a health service perspective will be taken, but does not specify the primary cost-effectiveness metric (e.g., cost per week of wait time reduced? cost per additional patient seen within target time?). Without this, the economic analysis is difficult to evaluate.

6. **Typographical Error:** The protocol lists two step "(iv)" instead of steps (iv) and (v). The fifth step is described as "implement tailored strategies to enhance patient flow." This minor error does not undermine the content but suggests a proofreading oversight.

Conclusion

This is a well-conceived, methodologically sound protocol that addresses a high-priority healthcare problem. The hybrid design is a particular strength. The main concerns are statistical power with only eight clusters and potential contamination. Nevertheless, if implemented as described (with the addition of a power calculation and contamination mitigation plan), the WaitLESS trial will provide valuable evidence for health systems seeking to reduce outpatient waiting times using demand-driven strategies.

Recommendation for peer review: Minor revision.

Suggestions for the Authors:

1. **Add a power calculation** and justify the number of clusters and observations.
2. **Describe strategies to reduce contamination** (e.g., using different physical locations, staggered training, or monitoring cross-cluster communication).
3. **Clarify the economic evaluation** – state the primary incremental cost-effectiveness ratio (ICER) and the willingness-to-pay threshold.
4. **Acknowledge the limitation** of few clusters and describe the planned sensitivity analyses (e.g., using permutation tests for the intervention effect).
5. **Correct the typographical error** in the step numbering.

VERSION 2 - AUTHOR RESPONSE

Response to reviewers – Revision 2

Thank you for providing a second review of this paper. We have provided

Comment 1.

Risk of Contamination: In a stepped wedge design within a single metropolitan health network, there is a risk that staff from clusters not yet randomized to start the STAT model might learn about the model from colleagues in active clusters, or that system-wide changes (e.g., a new electronic referral system) could affect all clusters simultaneously. The paper does not mention how contamination will be mitigated.

Response:

Contamination is an important point for consideration and we agree that there is some risk of contamination between clusters. As noted in the response to the original review of this paper, this is one of the realities of health services research of this nature, where interventions are implemented in large organisations and it is not possible to control all aspects of the intervention environment. This risk is being mitigated by (1) including a diverse range of clinical specialties which minimise the likelihood of the same doctors working across more than one cluster; and (2) having a structured implementation process to guide the nature and timing of

changes to clinic operations. The Design section (page 6) (modified in response to a similar comment raised in the first review of our paper) acknowledges this issue:

> *While the design carries some risk of contamination and implementation fatigue due to the possibility of some of the same staff working across different clusters, this will be minimised through inclusion of a diverse range of clinical specialties and structured implementation process…"*

This issue will also be noted as a limitation of the trial when results are reported.

In terms of system wide changes, such as a new electronic referral system, stepped wedge designs provide some protection against this type of bias. By nature of the design, any confounders like this will occur in pre-implementation for some clusters, and mid or post implementation for others. In addition, we plan to conduct within-cluster sensitivity analyses using interrupted time series analysis to explore the possible impact of events unrelated to the trial. This is explained under the analysis section on page 15:

> *In the event of any system-wide (e.g. pandemic) and within-cluster notable disruptions (e.g. staffing issues) that are not adequately captured by the baseline random-effects structure and residual variation, a disruption indicator variable will be used for the disrupted time period. This model of adjustment to disruption can also be included as an indicator within affected clusters only. As an additional sensitivity analysis, within-cluster time-series analyses will be conducted to account for localised temporal disruptions while assessing the persistence of intervention effects.*

Comment 2.

Statistical Power & Clustering: The analysis plan mentions linear mixed effects models, which is correct for clustered data. However, with only eight clusters (specialties), the study may have limited power to detect a modest effect size, especially if there is high intracluster correlation (i.e., waiting times vary more between specialties than within specialties over time). The paper does not report a sample size or power calculation.

Response:

A thorough power calculation is included, and was situated in paragraph 1 of the section *"1. Effectiveness"* under analysis on page 14. To improve clarity of the manuscript, this information regarding sample size has been moved under a separate heading "sample size" prior to the "analysis" section, which now reads:

> ***Sample size***
>
> *A minimum average of 400 referrals is expected to be received for each included specialty annually (100 per quarter), resulting in at least 700 patients across the pre- and post-implementation periods for each cluster (minimum total sample, n=5,600). With four steps and eight clusters (two clusters per step), this stepped wedge design will be well powered to detect even small to moderate effect sizes. For a mean difference of 0.25 standard deviations in waiting time and a conservative ICC of 0.1, with at least 60*

referrals per cluster per three-month time period (which is less than the pro-rata expected number annually), this design is powered at approximately 90% to sufficiently detect a difference at a 5% level of significance.(33, 34) Again, under conservative assumptions, power for detecting significant differences for our secondary outcomes (e.g. an increase in the proportion of urgent patients seen within 30 days from 80 to 90%) exceeds 90%. Compared to our findings from STAT implementation in community outpatient services,(23) our power and sample size assumptions are chosen conservatively to more than compensate for additional heterogeneity or other differences in the specialised clinic setting.

The sample size for quantitative implementation outcomes is based on the duration of the intervention and length of the waiting lists of the included services. The sample size for the qualitative analysis is considered feasible and expected to be sufficient to reach information power.

This power calculation demonstrates that the sample size is sufficient to detect meaningful change.

Comment 3.

Unit of Analysis Issue: The primary analysis appears to be at the patient level (referral to appointment time), but randomization is at the cluster level. This is statistically appropriate with mixed models, but the paper does not specify how degrees of freedom for the intervention effect will be handled (e.g., using cluster robust standard errors or Kenward-Roger approximation), which is critical with few clusters.

Response

This is a good point that we agree warrants inclusion in the protocol. The Kenward-Roger small-sample correction will be used for the degrees of freedom. The following sentence has been added to the analysis section of the protocol.

Given the small number of clusters, the Kenward-Roger small-sample correction will be used for the degrees of freedom for inference of the fixed effects.

Comment 4

Step Duration: A three-month interval between cluster starts is relatively short. Given the six-month pre-implementation and six-month follow-up, it is unclear if this allows sufficient time for each specialty to complete all five STAT steps (especially step iii: address existing backlogs) before the next clusters begin. Implementation fatigue could be an issue.

Response

The design is not intended to allow each clinic to fully complete implementation before the next cluster begins. Overlap between intervention periods is a common feature of stepped wedge designs requiring long implementation periods (eg see Watson SI, Hooper R. Grand rounds in

methodology: improving the design of staggered implementation cluster randomised trials. BMJ Qual Saf. 2025 Aug 18;34(9):631-636, Figure 1c).

The clinical teams introducing the intervention are mostly independent from one another (the small risk of contamination due to overlaps in staff was addressed in the response to the first review of this paper) and are therefore unlikely to be significantly impacted by implementation fatigue.

However, a minor modification has been made to the design section to acknowledge the potential risk of implementation fatigue and how it will be mitigated:

> *While the design carries some risk of contamination or implementation fatigue due to the possibility of some of the same staff working across different clusters, this will be minimised through inclusion of a diverse range of clinical specialties and structured implementation process supported by project staff.*

Comment 5

Missing Key Details on Cost-Effectiveness: The paper states a health service perspective will be taken, but does not specify the primary cost-effectiveness metric (e.g., cost per week of wait time reduced? cost per additional patient seen within target time?). Without this, the economic analysis is difficult to evaluate.

Response:

A minor change has been made to the wording in the health economic analysis section to ensure that the primary cost effectiveness metric is clear:

> *To establish the incremental cost-effectiveness ratio (ICER), pre-implementation to post-implementation, cost differences will be divided by the percentage difference of urgent and routine referrals seen within 30 and 90 days respectively to determine the cost per 1% increase in the proportion of patients seen within these thresholds.*

Comment 6. Typographical Error: The protocol lists two step "(iv)" instead of steps (iv) and (v). The fifth step is described as "implement tailored strategies to enhance patient flow." This minor error does not undermine the content but suggests a proof-reading oversight.

> *Thank you. This error has been corrected.*
